# Supplementary material for: Knowledge, attitude and risky practices on schistosomiasis in Ethiopia: A scoping review
Source: PLoS One. 2025 Nov 14;20(11):e0337085. doi: 10.1371/journal.pone.0337085 (PMC12617891; doi:10.1371/journal.pone.0337085)
Supplement: S3 File — (DOCX) [file pone.0337085.s003.docx]

| Quality appraisal was assessed using a 10-point scoring system quality assessment tool for cross-sectional studies  The total quality score varied between 0 and 10 where 1-4 = (Low); 5-7 = (Moderate) and 8-10=(High) | | | | | | | | | | | |
| --- | --- | --- | --- | --- | --- | --- | --- | --- | --- | --- | --- |
| Introduction | | **Methods** | | | | | **Results** | | **Discussions** | | **TOTAL** |
| Study citation | (1) clear definition of objectives/ aims | (2) study design appropriate for the stated aims | (3) sample size justified | (4) target population clearly defined (appropriate population base/unbiased sampling) | (5) risk factor and outcome variables measured correctly using instruments that had been trialled, piloted or published previously | (6) methods (including statistical methods) sufficiently described to enable them to be repeated | (7) results for analysis described in the methods, presented | (8) authors discussions and conclusions justified by results | (9) limitations of the study discussed | (10) ethical approval or consent of participants attained | Scores  (0-10) |
| [17] | 1 | 1 | 0 | 0 | 1 | 0 | 1 | 1 | 0 | 1 | 6 |
| [18] | 1 | 1 | 1 | 1 | 1 | 1 | 1 | 1 | 0 | 1 | 9 |
| [19] | 1 | 1 | 1 | 1 | 0 | 1 | 1 | 1 | 1 | 1 | 9 |
| [20] | 1 | 1 | 1 | 1 | 1 | 0 | 1 | 0 | 1 | 1 | 8 |
| [21] | 1 | 1 | 1 | 0 | 0 | 1 | 1 | 1 | 0 | 1 | 7 |
| [22] | 1 | 1 | 0 | 1 | 1 | 1 | 1 | 1 | 0 | 0 | 7 |
| [23] | 0 | 1 | 0 | 0 | 0 | 1 | 1 | 1 | 1 | 0 | 5 |
| [24] | 0 | 1 | 1 | 1 | 0 | 1 | 1 | 1 | 0 | 1 | 7 |
| [25] | 1 | 1 | 0 | 1 | 0 | 0 | 1 | 1 | 0 | 1 | 6 |
| [26] | 0 | 1 | 1 | 0 | 1 | 1 | 1 | 1 | 1 | 1 | 8 |
