# Supplementary material for: Knowledge, attitude and risky practices on schistosomiasis in Ethiopia: A scoping review
Source: PLoS One. 2025 Nov 14;20(11):e0337085. doi: 10.1371/journal.pone.0337085 (PMC12617891; doi:10.1371/journal.pone.0337085)
Supplement: S4 File — (DOCX) [file pone.0337085.s004.docx]

| **Name of data extractors (extraction date)**  Table: Extracted Data | **Confirmation of Eligibility of the study** | **Author/year** | **Study objectives** | **Type of study** | **Population/study location** | **Main findings** |
| --- | --- | --- | --- | --- | --- | --- |
| GA (03JAN2024)  EN (03JAN2024) | Confirmed | Mengstu *et al.,* 2009 [17] | To assess the level of awareness about intestinal Schistosomiasis in communities living in intestinal schistosomiasis endemic areas of Ethiopia | Cross-sectional study using open and closed-ended questionnaire | 417 community members  (176 from Dudicha (aged 15-70) and 241 from Shesha Kekel (aged 15-80) peasant associations) | - 123 (29.5%) heard about bilharzia - 96 (78%) suggested water as source of infection - Other sources of infection mentioned: poor sanitation (2, 1.6%), contagious (2, 1.6%), air (1, 0.8%), mosquito bite (1, 0.8%), do not know (21, 17.1%) - 62 (50.4%) mentioned water contact as way of transmission - 49 (39.8%) understood that bilharzia can be transmitted by both drinking contaminated water and during bathing or swimming - 53 (43.1%) knew abdominal pain or bloody diarrhea is a common symptom - 96 (78%) knew that bilharzia has treatment - 27 (22%) had no information whether bilharzia has treatment - 108 (87.8%) knew that the disease affects children and adults as well as males and females - 3 individuals from Shesha Kekel mentioned only children would catch bilharzia - 12 (9.8%) individuals were not sure which age groups are affected - 7 (5.7%) have knowledge about the intermediate host - 2 (0.83%) from Shesha Kekel mentioned bilharzia as a common disease in the area - 82(66.7%) knew that it is possible to prevent bilharzia by giving treatment, using clean water for drinking and washing and reducing water contact - 94/96 (97.9%) practice frequent water contact |
| GA (03JAN2024)  EN (03JAN2024) | Confirmed | Nyanteki *et al*., 2010 [25] | To assess the mothers’ (having <5 children) awareness about the cause, effect, mode of transmission, and preventive methods | Cross-sectional study using open-ended questionnaire | 130 mothers of PSAC from Shesha kekel | **Mothers’ response when asked ‘How do children get bilharzia?’**  Drinking dirty/river water (19, 16.4%)  Washing in river water (8, 6.9%)  Bad air, contaminated food or poor sanitation (13, 11.2%)  Do not know (82, 70.7%) |
| GA (05JAN2024)  EN (05JAN2024) | Confirmed | Nyanteki *et al*., 2014 [18] | To assess the knowledge of Abay Deneba village community | Community based cross-sectional study using structured and open ended questionnaire | 345 Household members | - Knowledge about the cause of SCH: contaminated water (64, 18.6%), contaminated food (4, 1.2%), don’t know (63, 18.3%), bath in river (5, 1.5%) - Knowledge about common symptoms: abdominal discomfort (13, 3.8%), back pain, headache (3, 0.9%) - Knowledge about preventive methods: avoid using contaminated water (49, 14.2%), don’t know (70, 20.4%) - 72.2% said their children had frequent contact with water bodies - 60% said their children bathed in different sources of water - 95.4% use water from Lake Ziway for drinking - 98% defecate in the open field - 89.3% did not receive health education |
| GA (07JAN2024)  EN (06JAN2024) | Confirmed | Alemu *et a*l., 2016 [26] | To assess the prevalence of *S.mansoni*, STH infections and  associated risk factors among preschool-aged children in  Denbia district, North West Ethiopia. | Community based cross-sectional study using questionnaire | 1. Mothers of PSAC | - 324 (80.8 %) had never heard about intestinal SCH - Major source of information was health professionals - 85 % responded that intestinal SCH cannot be transmitted by swimming or bathing in the river, crossing river with bare foot, washing clothes in river and fishing in river - 380 (94.8 %) of mothers did not know ways of prevention of intestinal SCH - 377 (94 %) did not know the symptoms of the disease |
| GA (07JAN2024)  EN (07JAN2024) | Confirmed | Gebreyohanns *et al*., 2018 [19] | To determine the prevalence of intestinal parasites and KAPs  among individuals who have river water contact with special emphasis on *S*. *mansoni* in the  town of Addiremets, Ethiopia. | Community based cross-sectional study using questionnaire | 301 HH members >15 years old | - 292 (97%) heard about SCH - Source of information: health facility (123, 43.4%), friends (82, 29%), school (67, 23.7%), radio (11, 3.9%) - When asked about ways of transmission: swimming in infested river water (132, 43.9%), drinking dirty water (171, 56.8%), playing in infested water (43, 14.3%), snail (18, 6%), contaminated food (35, 11.6%), don’t know (36, 12%) - When asked about the sign and symptoms: fever (61, 20.3%), headache (8, 2.7%), weakness (81, 26.9%), dry cough (1, 0.3%), abdominal pain (155, 51.5%), diarrhea (102, 33.9%), blood in stool (79, 26.2%), do not know (87, 28.9%) - 293 (97.3%) knew that SCH is treatable - 283 (94%) knew that SCH is preventable - When asked about prevention methods: treatment (54, 19.1%), Avoid bathing or swimming in stagnant water (128, 45.2%), use of toilet (64, 22.6%), provision of safe water (198, 70%), avoid open defecation (13, 4.6%), personal hygiene (13, 4.6%), do not know (1, 0.4%). - 289 (96%) believe SCH is a serious disease - 300 (99.7%) believe medication against SCH is important - 258 (85.7%) think swimming/bathing in river water can cause SCH - 296 (98.3%) think SCH is treatable - 279 (92.7%) wash clothes in river - 265 (88%) swim/bath in river - 170 (56.5%) defecate around rivers - 29 (9.6%) fetch river water for drinking/cooking |
| GA (09JAN2024)  EN (10JAN2024) | Confirmed | Mohammed *et al*., 2018[20] | To assess community awareness of *S. mansoni* in Haradenaba and Dertoramis kebeles in the Bedeno district, eastern Ethiopia. | Community based cross-sectional study using structured questionnaire | 1. participants of age ≥18 | - 466 (81.4% ) did not know at risk population groups for *S. mansoni* - 452 (79%) did not know the possible sources of infection - 494 (86.4%) did not know the possible modes of transmission - 452 (79%) did not know sign and symptoms - 452 (79%) did not know if SCH is treatable - 452 (79%) did not know if SCH is preventable |
| GA (10JAN2024)  EN (10JAN2024) | Confirmed | Assefa *et al*., 2021[21] | To investigate the levels  of schistosomiasis related to KAP of schistosomiasis endemic  community in the remote lowlands of the Abbey and Didessa Valleys in Benishangul Gumuz Region | A cross-sectional multilevel triangulation-mixed methods | 376 participants | - 328 (87.2%) never heard of SCH. 48 (12.8%) heard about SCH - Main sources of information were family or friends (45.8%) and schools (36.7%), health facilities (20.4%). - When asked about causes of SCH said that the cause for SCH is worm biting (62.5% , 30/48), drinking dirty water (8.3%, 4/48), eating food long time after cooked (6.3%, 3/48), did not know (22.9%, 11/48) - 73% (35/48) knew the ways of SCH transmissions - 88.6% (31/35) said while swimming/bathing in water bodies, 62.9% (22/35) said contact with worm and 22.9% (8/35) said drinking contaminated water. - 39.6% (19/48) knew the symptoms of schistosomiasis and all of them mentioned itching as the most common symptom, blood in stool (5.3%), abdominal pain (42.1%), swollen abdomen (5.3%) - 81.2% did not know ways of prevention. 9 (18.8%) knew it is preventable - Prevention methods: avoiding contact with water bodies (6/9), MDA (7/9) - 22.9% (11/48) agree that they were at the risk of *Schistosoma* spp. infection during contact with river or stream water bodies - 2.1% (1/48) believed SCH is a serious disease, 93.7% did not know if it is serious - 10.4% agree SCH is preventable - 0% believe human excreta is not a source of infection - Among 109 participants who were aware of MDA, 73 (67%) agree with its importance, but only 3 (2.8%) agree that MDA is effective - Majority cross water bodies on the way to school or work - 38.6% had gone to river/stream at least once per day to swim/bath or fetch water - 87.5% said they have latrine but rivers and stream sides were full of human excreta |
| GA (12JAN2024)  EN (11JAN2024) | Confirmed | Meleko *et al*., 2023 [24] | To assess the prevalence of SCH and STH and examine the association between these diseases KAP of schoolchildren in the newly established Gidi Bench district, of Bench Sheko Zone, Southwest Ethiopia | School based cross sectional study | 611 SAC | - 302 (49.4%) heard about intestinal parasitosis - When asked about how SCH is transmitted: eating contaminated food (23.7%, 145), dirty hands (16.2%, 99), Swimming or bathing in infested water (16.7%, 102), playing with soil (20%, 122) - 40.8%% (249) swim/bath/play in river - 59.4% (363) wash clothes or utensils in open water sources |
| GA (17JAN2024)  EN (17JAN2024) | Confirmed | Sule *et al*., 2022 [22] | To evaluate theatre‑based behavior change approach for influencing community uptake of SCH control measures | Community based follow up study | 369 participants | - 75% knew SCH affect both children and adults - 50% and 44% knew SCH is very dangerous and dangerous, respectively - 95% believe health facility is the right place to get treatment |
| GA (23JAN2024)  EN (23JAN2024) | Confirmed | Micho *et al*., 2020 [23] | To assess the magnitude of intestinal parasites among Zay people residing in three islands of Lake Ziway in Ethiopia. | Community based Cross-sectional study | 444 people in Ziway | - 31% never heard about bilharziasis. - 36% had no information about waterborne and related diseases - 85.6% use lake water for cleaning, drinking or both |

Note: data from each study was independently extracted by GA (Getaneh Alemu) and EN (Endalkachew Nibret), and discrepancies were solved by discussion
