# Supplementary material for: Knowledge, attitude and risky practices on schistosomiasis in Ethiopia: A scoping review
Source: PLoS One. 2025 Nov 14;20(11):e0337085. doi: 10.1371/journal.pone.0337085 (PMC12617891; doi:10.1371/journal.pone.0337085)
Supplement: Attachment — Submitted filename: General Reviewer Observations and Comments.pdf [file pone.0337085.s006.pdf]

## General Reviewer Observations and Comments

A systematic review on the knowledge, attitude, and practice (KAP) regarding schistosomiasis in Ethiopia reveals several critical insights. Generally, knowledge about schistosomiasis is variable, often influenced by factors such as education, geographic location, and access to health information.

Many communities have limited understanding of the transmission dynamics, symptoms, and prevention strategies associated with the disease. This gap in knowledge frequently translates to negative attitudes towards preventive measures, with some cultural beliefs affecting perceptions of the disease.

Moreover, behavioral practices associated with schistosomiasis prevention, including safe water usage and sanitation, are often inadequate.

Lack of access to clean water and proper sanitation facilities exacerbates the situation, contributing to the persistence of the disease in endemic regions.

**Comments for review**

- There is a lot of systematic editing and reviewing required from the investigator.
- When conducting research or a systematic review on knowledge, attitude, and practice (KAP) regarding schistosomiasis, various statistical analysis methods and data presentation tools should be used which are missing in the Data Analaysis section:
- Gaps in statistical analysis methods and the team could have used one of the following:
  - Descriptive Statistics: Mean, Median, Mode: To summarize central tendencies in knowledge scores or attitudes.
  - Frequency and Percentage: To detail respondents' knowledge levels, attitudes, and practices concerning schistosomiasis.
  - Inferential Statistics: Chi-Square Test: To examine associations between categorical variables (e.g., knowledge levels across different demographic groups).
  - T-Tests or ANOVA: To compare means of knowledge or attitude scores between different groups, such as educational levels or regions.
  - Correlation Analysis: To assess relationships between knowledge and practice scores using Pearson or Spearman correlation coefficients.
  - Regression Analysis: To identify factors influencing practices regarding schistosomiasis (e.g., logistic regression for binary outcomes).

- Factor Analysis: To identify underlying relationships between different KAP components and determine key factors affecting knowledge and practices.

Gaps in Data Presentation/Graphs/Charts and the team could have used one of the following:

- Tables: For presenting descriptive statistics, frequency distributions, and results of inferential analyses clearly.
- Bar Charts: To represent categories of knowledge and attitudes visually.
- Pie Charts: For displaying proportions of respondents' attitudes or practices.
- Box Plots: To show distributions of knowledge scores across different groups.
- Geospatial Analysis Tools: If location data is available, tools like ArcGIS can visualize schistosomiasis prevalence and KAP data across different geographic areas.

Using these statistical methods and presentation tables would have enhanced the clarity and impact of the findings related to KAP on schistosomiasis in Ethiopia.
