# Supplementary material for: Knowledge, attitude and risky practices on schistosomiasis in Ethiopia: A scoping review
Source: PLoS One. 2025 Nov 14;20(11):e0337085. doi: 10.1371/journal.pone.0337085 (PMC12617891; doi:10.1371/journal.pone.0337085)
Supplement: Attachment — Submitted filename: Manuscript-KAP review final.docx [file pone.0337085.s007.docx]

**Knowledge, attitude and practice on schistosomiasis in Ethiopia. A systematic review**

Getaneh Alemu^1*^, Endalkachew Nibret^2, 3^, Abaineh Munshea^2, 3^, Melaku Anegagrie^4^, Arancha Amor^4^

**Affiliations**

^1^Department of Medical Laboratory Science, Bahir Dar University, Bahir Dar, Ethiopia

^2^Biology Department, Science College, Bahir Dar University, Bahir Dar, Ethiopia

^3^Health Biotechnology Division, Institute of Biotechnology (IoB), Bahir Dar University, Bahir Dar, Ethiopia

^4^Mundo Sano Foundation and Institute of Health Carlos III, Madrid, Spain

^*^corresponding author

Email address of corresponding author: [getanehmlt@gmail.com](mailto:getanehmlt@gmail.com)

Cell phone: +251922842776

PO. BOX: 79

**Abstract**

**Background:** Health education and promotion enable the involvement and active participation of the community in disease control and prevention. Therefore, it is one of the key strategies for the prevention and control of schistosomiasis (SCH) in Ethiopia. However, comprehensive data on the knowledge, attitude, and practice (KAP) among at-risk population groups towards the disease are lacking.

**Methods:** A systematic search of studies published between 2006 and 2023 in Ethiopia was done from PubMed, Hinari, African journal online and Google Scholar using key words: “schistosomiasis, *Schistosoma*, *Schistosoma mansoni*, *Schistosoma haematobium*, knowledge, attitude, practice, awareness, perception, belief, Ethiopia” by combining with Bolean operators (AND, OR). Eligible studies were selected and reviewed following the PRISMA guidelines and checklist. Thematic analysis was utilized for the synthesis of the selected studies.

**Results:** Knowledge gaps about the source of *Schistosoma* infection, transmission, morbidity, treatment, and prevention in Ethiopia were identified. Great attitude variations were found across studies towards SCH in terms of population at risk, the seriousness of the disease, and beliefs in the availability and success of its treatment and prevention. Moreover, in most studies included in this review, the majority of the participants had negative attitude towards SCH. Majority of participants also involved in risky water-related practices, promoting the persistent transmission of SCH. The KAP level among community members, school-aged children, and mothers/caregivers of pre-school-aged children did not show significant variation.

**Conclusions:** Findings in this systematic review showed inadequate KAP, despite health education platforms that have been established and implemented for many years. Therefore, we recommend strengthening the implementation of health education and continuous monitoring of SCH prevention and control activities.

**Keywords:** schistosomiasis, knowledge, attitude, practice, Ethiopia, systematic review

**Background**

Schistosomiasis (SCH) is one of the 20 neglected tropical diseases (NTDs), and it is caused by trematodes of the genus *Schistosoma* [1]. The disease is endemic in 70 developing countries, and more than 200 million people are infected worldwide. *Schistosoma mansoni* and *S. haematobium* are the most prevalent species both globally and in Africa. Sub-Saharan Africa (SSA) carries the highest burden of SCH cases (90% of the infections), and up to 20 million people suffer from severe chronic health consequences of the disease. The disability-adjusted life years lost due to SCH are estimated to be 3.3 million [2].

Ethiopia is one of the countries in SSA having the largest burdens of NTDs, including SCH [3]. Between the year 2015 and 2020, a total of 966 districts were mapped. Out of these, 480 were endemic for SCH, and about 53.3 million people were at risk of infection. Both the intestinal (caused by *S. mansoni*) and urogenital (caused by *S. haematobium*) forms of SCH are prevalent in Ethiopia [4]. Cognizant of this fact, the Federal Ministry of Health (FMoH) had launched the first NTDs control program and implemented it between 2013 and 2015 [5]. During this period, 81.3% (10 million out of 12.3 million) of targeted school-aged children (SAC) were dewormed with praziquantel. However, the absence of adequate attention to social behavioral change communication (SBCC), poor clean water supply, and low level of sanitation and hygiene practices are among the major challenges to sustaining the interventions. The 2^nd^ national NTD strategic plan which aimed to eliminate SCH to a level where it is no longer a public health problem by 2020’was implemented from 2016 to 2020. Although 27 million SAC were treated during this period, SCH is still a public health problem. Currently, Ethiopia is implementing the 3^rd^ national NTD strategic plan (2021-2025) which aims to eliminate *Schistosoma* transmission in Ethiopia by 2025. Mass drug administration (MDA), facility-based case diagnosis and treatment, snail control in hotspots, SBCC, and access to safe water supply, basic sanitation, and hygiene (WASH) are the five strategies identified to achieve this goal [4]. In addition to the national NTD strategic plans, the health extension program, which became operational in Ethiopia in 2004-2005 and is being implemented, was intended to involve the community in disease prevention through the transfer of health knowledge and skills to households [6]. However, preventable diseases like SCH, soil-transmitted helminthes (STHs), and malaria continue to be major public health problems [4, 7].

Finally, several studies have shown that reinfection after MDA has been the main reason for persistent *Schistosoma* spp. transmission in many endemic areas [4, 8, 9]. Hence, MDA should be implemented in integration with SBCC, WASH, and vector control programs. Although SAC, women, and other people whose occupation is linked to contacting natural water bodies are at a greater risk of *Schistosoma* spp. infection [10, 11], the intervention programs primarily target SAC. Schistosomiasis prevention targeting all populations at-risk would have enabled the successful control of the disease [12].

Successful implementation of SCH control and prevention strategies demands engagement and active participation from the community. Therefore, ideally, health education should precede programs that require the full involvement of the community, as this will enable the community to make informed decisions regarding their participation [13]. In fact, in cognizant of this, the World Health Organization (WHO) as early as in 1985 suggested health education and health promotion to be the focus of SCH prevention work [14]. However, a systematic review of articles published between 2006 and 2016 in the SSA showed that the KAP level varied between studies [15]. Even though, a few studies were conducted in Ethiopia, the previous review did not include them with a possible reason of robust eligibility criteria and review period.

Health education has been taken as one of the key strategies for the control and elimination of SCH in previous Ethiopian national NTD strategic plans. However, changes in the population’s KAP towards SCH have not been well addressed. Even the existing information is fragmented, and there is no updated comprehensive nationwide data in this regard. Hence, the aim of this review was to assess KAP towards SCH among different population groups in Ethiopia. This systematic review will help stakeholders to evaluate the impact of the ongoing health education programs.

**Methods**

**Search strategy**

We conducted a search of articles published between January 2006 and December 2023. Our aim was to assess the progress in the KAP level towards SCH in Ethiopia after the start of the health extension program (2004-2005). Two independent reviewers (GA and EN) searched literatures in the databases Pubmed, Hinari, African journal online, and Google Scholar, using the key words “schistosomiasis, *Schistosoma*, *Schistosoma mansoni*, *Schistosoma haematobium*, knowledge, attitude, practice, awareness, perception, belief, Ethiopia.” Searches were made by starting with broad terms and narrowing step by step, and combining key words with Bolean operators (AND, OR). Additional searches were made based on the references listed in the identified publications. We conducted and reported this review following the PRISMA (Preferred Reporting Items for Systematic Reviews and Meta-Analyses) guideline (Fig. 1).

**Study selection**

Study selection was made in a three-stage review process: database search, screening of abstracts by two authors, and eligibility checking, as shown in the PRISMA flow diagram. Studies conducted in Ethiopia between 2006 and 2023 that focused on the KAP of any population, towards either intestinal or urogenital schistosomiasis, and published in English were included in the present study. Studies published before 2006 were excluded. Selected papers were read thoroughly to find the best available data to fulfill the objective of this review.

**Quality assessment**

A quality appraisal of the included studies was made using a critical appraisal tool for the assessment of cross-sectional studies adapted from Downes Brennan *et al*. [16]. Studies were evaluated based on the following indicators: (1) clear definition of objectives; (2) study design appropriate for the stated objectives; (3) sample size justified; (4) target population clearly defined (appropriate population base/unbiased sampling); (5) risk factor and outcome variables measured correctly using instruments that had been trialed, piloted, or published previously; (6) methods (including statistical methods) sufficiently described to enable them to be repeated; (7) results for analysis described in the methods presented; (8) author discussions and conclusions justified by results; (9) limitations of the study discussed; (10) ethical approval or consent of participants. Answers for each indicator were scored 0 for ‘No’ and 1 for ‘Yes’. The quality of papers was declared low, moderate, and high if the total scores were between 1-4, 5-7 and 8-10, respectively (Supplementary file 1). Accordingly, six and four studies were with moderate and high quality, respectively.

**Data extraction**

We extracted data about the first author, publication year, study objective, study design, study population, study location, and main study findings into a table. A thematic analysis was utilized for the overall synthesis of the selected studies.

**Results**

A total of 1310 articles were retrieved from all searched databases and webs. Six hundred forty-four articles were removed as they were duplicates. Additional 650 articles were removed after reading the titles and abstracts due to the following reasons: 1) irrelevant to the review title (582); 2) conducted outside Ethiopia (64); 3) reviews (3); 4) conducted before 2006 (2). A further six were excluded because we could not retrieve full texts. Hence, 10 articles were included in the present review. After a detailed reading of each included study, four themes were identified: 1) sociodemographic characteristics; 2) knowledge about SCH; 3) attitude related to SCH; 4) practices related to SCH prevention and control. Seven studies were community-based cross-sectional studies [17-23]; one study was conducted from SAC [24], while the remaining two studies recruited women having pre-school-aged children (PSAC) [25, 26]. Three studies were conducted before the start of the national school-based MDA in 2015 [17, 18, 25], while data for the remaining seven studies were collected after the start of the MDA [19-24, 26]. Studies included in the present review recruited 4,763 participants (2,118 male and 2,645 female) with ages ranging from 5 to 86 years old [17-20, 22-26], despite, one study did not specify the maximum age limit [21]. Data on KAP related to SCH were collected by administering a structured questionnaire, except in one study where a focus group discussion was conducted [21]. The ten reviewed studies are summarized in Table 1.

**Knowledge about schistosomiasis**

Six out of the ten studies reported the proportion of respondents who had ever heard of the disease SCH/bilharziasis. In four articles, the majority of respondents: 87.2% [21], 80.8% [26], 71.5% [17] and 51.6% [24] had never heard about SCH. Two studies reported a high proportion of awareness among participants, with 97% [19], and 69% [23] having heard about the disease. Three studies reported the sources of information, being health facilities/health professionals, family/friends and schools the primary ones for most of the participants [19, 21, 26].

In most studies, there was poor knowledge about the source and modes of infection, and the majority of the interviewees responded “I don’t know” when asked about both issues [17-20, 25, 26]. Related to the source of SCH infection, three studies reported good knowledge, as the majority of the respondents mentioned contaminated water [17, 21, 26], while three other reported poor knowledge [18, 23, 25]. About the modes of SCH transmission, adequate knowledge was reported in only one study, in which 88.6% (31/35) of participants responded that swimming or bathing in contaminated water exposed them to infection [21].

Among participants who were aware of SCH, 28.9% [19], 50.4% [17], 60.4% [21], 79% [20], and 94% [26] did not know at least one sign or symptom of SCH. Moreover, most of the participants who knew the signs and symptoms were unable to comprehensively list the common signs and symptoms. Respondents were asked, “Is schistosomiasis treatable?” in four studies. The proportion of participants who correctly responded were 1.7% [25], 21% [20], 29% [21], 78% [17], and 97.3% [19] among participants who had ever heard of SCH. In three studies, the majority of the interviewees - 66.7% [17], 79.6% [18], and 72.1% [19] - knew that SCH is preventable, while the other three studies reported poor knowledge, as 79% [20], 87.2% [21], and 94% [26] of the respondents did not know whether SCH can be prevented or not.

**Attitude towards schistosomiasis**

A significant number of interviewees believed that drinking dirty water, eating contaminated food, air, infected mosquitoes, and flies are sources of *Schistosoma* infection [17-19, 24, 25]. In one study, 4

(1.2%) participants believed that contaminated food was a source of infection, and 2(0.6%) believed cooking vegetables and hygienic practices prevented SCH [18]. Another study reported that participants believed eating contaminated food (23.7%, 145), dirty hands (16.2%, 99), and playing with soil (20%, 122) predispose them to *Schistosoma* infection [24]. In one of the articles, 85% of respondents believed that intestinal SCH could not be transmitted by swimming or bathing in river, crossing river with bare foot, washing clothes in river or fishing in river [26]. In another study, only 22.9% (11/48) of interviewees agreed that they were at risk of *Schistosoma* infection during their contact with river or stream water. In the same study, the majority 93.7% (45/48) said that they were not sure whether SCH was a serious disease or not [21]. However, in two studies, 96% (289/301) [19] and 94% [22] of the participants understood SCH as a serious disease. Related to treatment and preventive chemotherapy, in one study, 67% (73/109) of the participants agreed with the importance of MDA, but only 2.8% (3/109) agreed that mass therapy was effective for SCH control [21]; in another study, about 99.7% agreed with the importance of medication during SCH [19]. Finally, the majority 94% [19] and 66.7% [17] of the participants who had the awareness agreed that SCH is preventable, while 89.6% [21] and 79% [20] in other studies did not agree with this fact.

**Risky practices for *Schistosoma* infection**

Many of the participants had frequent freshwater contact while swimming or bathing, washing clothes and utensils, crossing rivers bare-foot or fetching freshwater for domestic use [17-19, 21, 23, 24]. In two studies, 98% [18] and 56.5% [19] of the participants who had the awareness frequently defecate in the open field and around rivers. In one study, 87.5% of the respondents said they have latrine, but rivers and stream sides in the study area were full of human excreta up on field observation [21]. Moreover, in one study, 89.3% said they had never received health education targeting SCH prevention [18].

**Table 1:** Summary of studies in this review

| **Author/year** | **Study objectives** | **Type of study** | **Population/study location** | **Main findings** |
| --- | --- | --- | --- | --- |
| Mengstu *et al.,* 2009 [17] | To assess the level of awareness about intestinal Schistosomiasis in communities living in intestinal schistosomiasis endemic areas of Ethiopia | Cross-sectional study using open and closed-ended questionnaire | 417 community members  (176 from Dudicha (aged15-70) and 241 from Shesha Kekel (aged 15-80) peasant associations) | - 123 (29.5%) heard about bilharzia - 96 (78%) suggested water as source of infection - Other sources of infection mentioned: poor sanitation (2, 1.6%), contagious (2, 1.6%), air (1, 0.8%), mosquito bite (1, 0.8%), do not know (21, 17.1%) - 62 (50.4%) mentioned water contact as way of transmission - 49 (39.8%) understood that bilharzia can be transmitted by both drinking contaminated water and during bathing or swimming - 53 (43.1%) knew abdominal pain or bloody diarrhea is a common symptom - 96 (78%) knew that bilharzia has treatment - 27 (22%) had no information whether bilharzia has treatment - 108 (87.8%) knew that the disease affects children and adults as well as males and females - 3 individuals from Shesha Kekel mentioned only children would catch bilharzia - 12 (9.8%) individuals were not sure which age groups are affected - 7 (5.7%) have knowledge about the intermediate host - 2 (0.83%) from Shesha Kekel mentioned bilharzia as a common disease in the area - 82(66.7%) knew that it is possible to prevent bilharzia by giving treatment, using clean water for drinking and washing and reducing water contact - 94/96 (97.9%) practice frequent water contact |
| Nyanteki *et al*., 2010 [25] | To assess the mothers’ (having <5 children) awareness about the cause, effect, mode of transmission, and preventive methods | Cross-sectional study using open-ended questionnaire | 130 mothers of PSAC from Shesha kekel | **Mothers’ response when asked ‘How do children get bilharzia?’**  Drinking dirty/river water (19, 16.4%)  Washing in river water (8, 6.9%)  Bad air, contaminated food or poor sanitation (13, 11.2%)  Do not know (82, 70.7%) |
| Nyanteki *et al*., 2014 [18] | To assess the knowledge of Abay Deneba village community | Community based cross-sectional study using structured and open ended questionnaire | 345 Household members | - Knowledge about the cause of SCH: contaminated water (64, 18.6%), contaminated food (4, 1.2%), don’t know (63, 18.3%), bath in river (5, 1.5%) - Knowledge about common symptoms: abdominal discomfort (13, 3.8%), back pain, headache (3, 0.9%) - Knowledge about preventive methods: avoid using contaminated water (49, 14.2%), don’t know (70, 20.4%) - 72.2% said their children had frequent contact with water bodies - 60% said their children bathed in different sources of water - 95.4% use water from Lake Ziway for drinking - 98% defecate in the open field - 89.3% did not receive health education |
| Alemu *et a*l., 2016 [26] | To assess the prevalence of *S.mansoni*, STH infections and  associated risk factors among preschool-aged children in  Denbia district, North West Ethiopia. | Community based cross-sectional study using questionnaire | 401 Mothers of PSAC | - 324 (80.8 %) had never heard about intestinal SCH - Major source of information was health professionals - 85 % responded that intestinal SCH cannot be transmitted by swimming or bathing in the river, crossing river with bare foot, washing clothes in river and fishing in river - 380 (94.8 %) of mothers did not know ways of prevention of intestinal SCH - 377 (94 %) did not know the symptoms of the disease |
| Gebreyohanns *et al*., 2018 [19] | To determine the prevalence of intestinal parasites and KAPs  among individuals who have river water contact with special emphasis on *S*. *mansoni* in the  town of Addiremets, Ethiopia. | Community based cross-sectional study using questionnaire | 301 HH members >15 years old | - 292 (97%) heard about SCH - Source of information: health facility (123, 43.4%), friends (82, 29%), school (67, 23.7%), radio (11, 3.9%) - When asked about ways of transmission: swimming in infested river water (132, 43.9%), drinking dirty water (171, 56.8%), playing in infested water (43, 14.3%), snail (18, 6%), contaminated food (35, 11.6%), don’t know (36, 12%) - When asked about the sign and symptoms: fever (61, 20.3%), headache (8, 2.7%), weakness (81, 26.9%), dry cough (1, 0.3%), abdominal pain (155, 51.5%), diarrhea (102, 33.9%), blood in stool (79, 26.2%), do not know (87, 28.9%) - 293 (97.3%) knew that SCH is treatable - 283 (94%) knew that SCH is preventable - When asked about prevention methods: treatment (54, 19.1%), Avoid bathing or swimming in stagnant water (128, 45.2%), use of toilet (64, 22.6%), provision of safe water (198, 70%), avoid open defecation (13, 4.6%), personal hygiene (13, 4.6%), do not know (1, 0.4%). - 289 (96%) believe SCH is a serious disease - 300 (99.7%) believe medication against SCH is important - 258 (85.7%) think swimming/bathing in river water can cause SCH - 296 (98.3%) think SCH is treatable - 279 (92.7%) wash clothes in river - 265 (88%) swim/bath in river - 170 (56.5%) defecate around rivers - 29 (9.6%) fetch river water for drinking/cooking |
| Mohammed *et al*., 2018[20] | To assess community awareness of *S. mansoni* in Haradenaba and Dertoramis kebeles in the Bedeno district, eastern Ethiopia. | Community based cross-sectional study using structured questionnaire | 572 participants of age ≥18 | - 466 (81.4% ) did not know at risk population groups for *S. mansoni* - 452 (79%) did not know the possible sources of infection - 494 (86.4%) did not know the possible modes of transmission - 452 (79%) did not know sign and symptoms - 452 (79%) did not know if SCH is treatable - 452 (79%) did not know if SCH is preventable |
| Assefa *et al*., 2021[21] | To investigate the levels  of schistosomiasis related to KAP of schistosomiasis endemic  community in the remote lowlands of the Abbey and Didessa Valleys in Benishangul Gumuz Region | A cross-sectional multilevel triangulation-mixed methods | 376 participants | - 328 (87.2%) never heard of SCH. 48 (12.8%) heard about SCH - Main sources of information were family or friends (45.8%) and schools (36.7%), health facilities (20.4%). - When asked about causes of SCH said that the cause for SCH is worm biting (62.5% , 30/48), drinking dirty water (8.3%, 4/48), eating food long time after cooked (6.3%, 3/48), did not know (22.9%, 11/48) - 73% (35/48) knew the ways of SCH transmissions - 88.6% (31/35) said while swimming/bathing in water bodies, 62.9% (22/35) said contact with worm and 22.9% (8/35) said drinking contaminated water. - 39.6% (19/48) knew the symptoms of schistosomiasis and all of them mentioned itching as the most common symptom, blood in stool (5.3%), abdominal pain (42.1%), swollen abdomen (5.3%) - 81.2% did not know ways of prevention. 9 (18.8%) knew it is preventable - Prevention methods: avoiding contact with water bodies (6/9), MDA (7/9) - 22.9% (11/48) agree that they were at the risk of *Schistosoma* spp. infection during contact with river or stream water bodies - 2.1% (1/48) believed SCH is a serious disease, 93.7% did not know if it is serious - 10.4% agree SCH is preventable - 0% believe human excreta is not a source of infection - Among 109 participants who were aware of MDA, 73 (67%) agree with its importance, but only 3 (2.8%) agree that MDA is effective - Majority cross water bodies on the way to school or work - 38.6% had gone to river/stream at least once per day to swim/bath or fetch water - 87.5% said they have latrine but rivers and stream sides were full of human excreta |
| Meleko *et al*., 2023 [24] | To assess the prevalence of SCH and STH and examine the association between these diseases KAP of schoolchildren in the newly established Gidi Bench district, of Bench Sheko Zone, Southwest Ethiopia | School based cross sectional study | 611 SAC | - 302 (49.4%) heard about intestinal parasitosis - When asked about how SCH is transmitted: eating contaminated food (23.7%, 145), dirty hands (16.2%, 99), Swimming or bathing in infested water (16.7%, 102), playing with soil (20%, 122) - 40.8%% (249) swim/bath/play in river - 59.4% (363) wash clothes or utensils in open water sources |
| Sule *et al*., 2022 [22] | To evaluate theatre‑based behavior change approach for influencing community uptake of SCH control measures | Community based follow up study | 369 participants | - 75% knew SCH affect both children and adults - 50% and 44% knew SCH is very dangerous and dangerous, respectively - 95% believe health facility is the right place to get treatment |
| Micho *et al*., 2020 [23] | To assess the magnitude of intestinal parasites among Zay people residing in three islands of Lake Ziway in Ethiopia. | Community based Cross-sectional study | 444 people in Ziway | - 31% never heard about bilharziasis. - 36% had no information about waterborne and related diseases - 85.6% use lake water for cleaning, drinking or both |

**Discussion**

This systematic review assessed knowledge, attitude, and practices on SCH among community members, SAC, and mothers/caregivers of PSAC in Ethiopia. Although the SCH control and prevention programs are designed centrally and thought to be implemented along with MDA all over the country [4], this review has shown variations in the KAP level across geographical settings and study populations. Apart from inadequate health education and promotion, socio-cultural factors might also be barriers to changes in attitude and practices towards SCH control and prevention [15].

According to this review, comprehensive knowledge about sources of infection, routes and modes of transmission, morbidity, treatment, and prevention of SCH is lacking in Ethiopia. This is unfortunate because health promotion on infectious diseases of public health importance has been delivered through the health extension program since 2004-2005 [6]. In addition, SCH has been identified as one of the five chemotherapy-preventable NTDs in Ethiopia. The chemotherapy has been accompanied by health education both in school and community settings [4]. The reduced knowledge level could be due to the inadequacy of health education sessions. For instance, we observed that school and community-based education sessions were conducted only at the time of deworming which is an inadequate method to establish good knowledge and change community behavior. If properly implemented, health education has a high input-output ratio and is a cost-effective prevention and control method. Having comprehensive knowledge contributes to having a positive attitude, which in turn contributes to behavioral changes like reducing risky activities [27]. However, behavioral change will come only if health education is accompanied by safe water supply and basic sanitation [28, 29]. This problem observed in this review, where participation in risky activities (frequent water contact) was high regardless of the participants’ knowledge level [17-26].

Misconceptions about the transmission and prevention of SCH, in addition to low knowledge level, might lead to poor prevention practices [30, 31]. In Ethiopia, MDA and health education programs both for SCH and STHs are implemented together. We understood that there is confusion regarding the transmission and prevention of SCH and STHs [17-19, 24, 25]. For instance, in one study, participants believed eating contaminated food (23.7%), dirty hands (16.2%) and playing with soil (20%) predispose for the infection [24]; although these actually predispose for STHs infection rather than SCH. Therefore, disseminating the right information specific to each disease during health education should be specially emphasized in programs including the control of more than one parasite (as it is STH together with SCH) and will be a key to enable people to apply appropriate preventive practices [32]. Misconceptions due to improper or mixed messages might contribute to wrong perceptions/beliefs among the community [33] which in turn affect their health seeking behaviors and participation in infection prevention [34].

Variations in the health education implementation at different transmission settings and study populations might contribute to differences in behavioral changes in Ethiopia. For example, the majority, 94% [19] and 66.7% [17] of participants who had the awareness agreed that SCH is preventable in two studies but 89.6% [21] and 79% [20] in other studies did not agree with this. Similarly, there were variations in attitude about the risk populations, seriousness, treatment, and prevention across studies [17-26].

The very purpose of health education is to provide knowledge about SCH and convince the community for their involvement in disease prevention and control. However, in the reviewed studies, majority of the participants involved in risky activities regardless of their knowledge and attitude towards SCH [17-26]. This might be due to failure to supplement health education with basic WASH facilities and health services [15].

**Limitations**

The proportion of participants with good knowledge, positive attitudes, and good preventive practices would have been quantified by the average scores of questionnaire items using Bloom’s modified cut-off values [35]. We were unable do this because of great variations of the items used to assess KAP, and hence, lack of standard definition of knowledge, attitude and practice. Therefore, interpretation of KAP results was subjective. This also hindered the comparison of KAP levels between the findings of studies. There was a great variation in the depth of data presented in reviewed studies such that studies presenting more data were over-represented in the review. The limited number of studies brought difficulty in driving definitive conclusion about the KAP level in Ethiopia.

**Conclusions**

Findings in the reviewed studies showed inadequate KAP level of people, despite both community and school-based health education platforms were established and implemented for more than a decade. This reminds for future strengthening of the health education program and monitoring of the practical implementation and impact assessment of the existing programs. Also, the implementation of two programs together (namely, STH and SCH), should be precise enough to avoid confusion and mixed information. Preparing a standard context-specific KAP assessment tool could be an immediate responsibility of the FMoH NTD prevention and control program. More studies are recommended both at school and community level to explore the KAP level and associated factors at different endemicity settings among all at-risk population groups.

**List of abbreviations**

BC	Behavioral Change

FMoH	Federal Ministry of Health

KAP	Knowledge Attitude and Practice

MDA	Mass Drug Administration

NTDs	Neglected tropical diseases

PSAC	Preschool-aged Children

SAC	School-aged Children

SBCC	Social Behavioral Change Communication

SCH	Schistosomiasis

SSA	sub-Saharan Africa

STHs	Soil-transmitted Helminths

WASH	Water Sanitation and Hygiene

WHO	World Health Organization

**Declarations**

**Ethics approval and consent to participate**

Not applicable.

**Human Ethics and Consent to Participate**

Not applicable.

**Consent for publication**

Not applicable.

**Availability of data and materials**

The original data for this study is available from the corresponding author.

**Competing interests**

The authors declare that they have no competing interests. All authors have seen and approved the final, submitted version of this manuscript.

**Funding**

No fund was obtained for execution of the review.

**Authors’ Contributions**

GA conceived and designed the review. GA and EN extracted data. GA, EN, AA MA, and AM analyzed data and reviewed the manuscript. GA wrote the manuscript. All authors read and approved the final manuscript.

**Acknowledgements**

Not applicable

**References**

1. WHO. Neglected tropical diseases. 2024. Available at: <https://www.who.int/news-room/questions-and-answers/item/neglected-tropical-diseases> (accessed 09 Jan 2024)
2. Global atlas of helminth infections. Available at: <https://www.thiswormyworld.org/worms/global-burden> (accessed 15 Dec 2023)
3. The End Fund. Neglected tropical diseases. Available at: <https://www.end.org/what-we-do/ethiopia> (accessed 04 Jan 2024)
4. Federal Democratic Republic of Ethiopia Ministry of Health (FMoH). The Third National Neglected Tropical Diseases Strategic Plan 2021-2025. 2021. Available at: [https://espen.afro.who.int/system/files/content/resources/Third NTD nationalStrategicPlan202021-2025.pdf](https://espen.afro.who.int/system/files/content/resources/Third%20NTD%20nationalStrategicPlan202021-2025.pdf) (Accessed 08 Feb 2022)
5. Mengitsu B, Shafi O, Kebede B, Kebede F, Worku DT, Herero M, *et al*. Ethiopia and its steps to mobilize resources to achieve 2020 elimination and control goals for neglected tropical diseases webs joined can tie a lion. *Int Health*. 2016; 8 (1): 34-52. doi: 10.1093/inthealth/ihw007
6. Banteyerga H. Ethiopia's health extension program: improving health through community involvement. MEDICC Rev. 2011; 13(3):46-9. doi: 10.37757/MR2011V13.N3.11.
7. WHO. World malaria report. 2023. <https://www.who.int/teams/global-malaria-programme/reports/world-malaria-report-2023> (Accessed 02 Jan 2024)
8. Zacharia A, Mushi V, Makene T. A systematic review and meta-analysis on the rate of human schistosomiasis reinfection. *PLoS One*. 2020; 15(12): e0243224. doi: 10.1371/journal.pone.0243224
9. Woldegerima E, Bayih AG, Tegegne Y, Aemero M, Jejaw ZA. Prevalence and Reinfection Rates of *Schistosoma mansoni* and Praziquantel Efficacy against the Parasite among Primary School Children in Sanja Town, Northwest Ethiopia. *J Parasitol Res*. 2019; 2019:3697216. doi: 10.1155/2019/3697216
10. Navas ALA, Hamm NA, Magalhães RJS, Stein A. Mapping soil transmitted Helminths and Schistosomiasis under uncertainty: a systematic review and critical appraisal of evidence. *PLoS Negl Trop Dis*. 2016; 10:e0005208. doi: 10.1371/journal.pntd.0005208
11. Manyangadze T, Chimbari MJ, Gebreslasie M, Mukaratirwa S. Risk factors and micro-geographical heterogeneity of Schistosoma haematobium in Ndumo area, uMkhanyakude district, KwaZulu-Natal, South Africa. *Acta Trop.* 2016;159:176–84. <https://doi.org/10.1016/j.actatropica.2016.03.028>
12. Gray DJ, McManus DP, Li Y, Williams GM, Bergquist R, Ross AG. Schistosomiasis elimination: lessons from the past guide the future. *Lancet Infect Dis*. 2010; 10:733–736
13. Gwatirisa PR, Ndamba J, Nyazema NZ. The impact of health education on the knowledge, attitudes and practices of a rural community with regards to schistosomiasis control using a plant molluscicide, Phytolacca dodecandra. *Cent Afr J Med*. 1999; 45(4): 94-97. doi: 10.4314/cajm.v45i4.8460
14. Feng J, Wang X, Zhang X, Hu H, Xue J, Cao C, *et al*. Effect of Health Education on Schistosomiasis Control Knowledge, Attitude, and Practice after Schistosomiasis Blocking: Results of a Longitudinal Observational Study in the Field. *Trop Med Infect Dis*. 2023; 8(5): 267. doi: 10.3390/tropicalmed8050267
15. Sacolo H, Chimbari M, Kalinda C. Knowledge, attitudes and practices on Schistosomiasis in sub-Saharan Africa: a systematic review. *BMC Infect Dis*. 2018; 18(1):46. doi: 10.1186/s12879-017-2923-6
16. Downes MJ, Brennan ML, Williams HC, Dean RS. Development of a critical appraisal tool to assess the quality of cross-sectional studies (AXIS). *BMJ Open*. 2016; 6(12): e011458. doi: 10.1136/bmjopen-2016-011458
17. Mengistu L, Chelsea RJ, Sarita KS, Berhanu E, Yalemtsehay M. Community’s awareness about intestinal schistosomiasis and the prevalence of infection in two endemic localities of Ethiopia. *Ethiop J Health Sci*. 2009; 19(2): 103-110
18. Nyantekyi L, Legesse M, Medhin G, Animut A, Tadesse K, Macias C, *et al*. Community awareness of intestinal parasites and the prevalence of infection among community members of rural Abaye Deneba area, Ethiopia. *Asian Pac J Trop Biomed*. 2014; 4(1):152-157. doi: 10.12980/APJTB.4.2014C764
19. Gebreyohanns A, Legese MH, Wolde M, Leta G, Tasew G. Prevalence of intestinal parasites versus knowledge, attitude and practices (KAPs) with special emphasis to *Schistosoma mansoni* among individuals who have river water contact in Addiremets town, Western Tigray, Ethiopia. *PLoS One*. 2018; 13(9): e0204259. doi: 10.1371/journal.pone.0204259
20. Mohammed J, Weldegebreal F, Teklemariam Z, Mitiku H. Clinico-epidemiology, malacology and community awareness of *Schistosoma mansoni* in Haradenaba and Dertoramis kebeles in Bedeno district, eastern Ethiopia. *SAGE Open Med*. 2018; 6: 2050312118786748. doi: 10.1177/2050312118786748
21. Assefa A, Erko B, Gundersen SG, Medhin G, Berhe N. Low awareness and common misconceptions about schistosomiasis in endemic lowland areas in Western Ethiopia: a mixed-methods study. *BMC Public Health*. 2021; 21(1):1064. doi: 10.1186/s12889-021-11106-y
22. Sule MN, Mosha J, Soboka TE, Kinung'hi SM, Sfynia C, Rafiq K, *et al*. A novel theatre-based behaviour change approach for influencing community uptake of schistosomiasis control measures. *Parasit Vectors*. 2022; 15(1):301. doi: 10.1186/s13071-022-05421-5
23. Micho H, Fantahun M, Gebreyohannes Z, Bedaso A, Bereka N, Abebayehu B, *et al*. High rate of intestinal parasites among a closed community of Zay populations residing on three islands of Lake Ziway, Ethiopia. *PLoS One*. 2020; 15(10): e0240582. doi: 10.1371/journal.pone.0240582
24. Meleko A, Turgeman DB, Caplan N, Baum S, Zerai NK, Zaadnoordijk W, *et al*. High prevalence of soil-transmitted helminths and schistosomiasis among primary schoolchildren in Southwest Ethiopia: the need for health strategies alongside mass drug administration. *Int Health*. 2023: ihad083. doi: 10.1093/inthealth/ihad083
25. Nyantekyi L, Mengistu L, Mulugeta B, Konjit T, Kebreten M, Chanda M, *et al*. Intestinal parasitic infections among under-five children and maternal awareness about the infections in Shesha Kekele, Wondo Genet, Southern Ethiopia. *Ethiop. J. Health Dev.* 2010; 24(3): 185-190
26. Alemu A, Tegegne Y, Damte D, Melku M. Schistosoma mansoni and soil-transmitted helminths among preschool-aged children in Chuahit, Dembia district, Northwest Ethiopia: prevalence, intensity of infection and associated risk factors. *BMC Public Health*. 2016; 16: 422. doi: 10.1186/s12889-016-2864-9
27. Asaolu SO, Ofoezie IE. The role of health education and sanitation in the control of helminth infections. *Acta Trop*. 2003; 86(2-3): 283-294. doi: 10.1016/s0001-706x(03)00060-3
28. Kabatereine N, Fleming F, Thuo W, Tinkitina B, Tukahebwa EM, Fenwick A. Community perceptions, attitude, practices and treatment seeking behaviour for schistosomiasis in L. Victoria islands in Uganda. *BMC Res Notes*. 2014; 7:900. <https://doi.org/10.1186/1756-0500-7-900>
29. Dawaki S, Al-Mekhlafi HM, Ithoi I, Ibrahim J, Abdulsalam AM, Ahmed A, *et al*. The menace of schistosomiasis in Nigeria: knowledge, attitude, and practices regarding schistosomiasis among rural communities in Kano state. *PLoS One*. 2015; 10:e0143667. doi: 10.1371/journal.pone.0143667
30. Tuhebwe D, Bagonza J, Kiracho EE, Yeka A, Elliott AM, Nuwaha F. Uptake of mass drug administration programme for schistosomiasis control in Koome Islands, Central Uganda. *PLoS One*. 2015; 10:e0123673. doi: 10.1371/journal.pone.0123673
31. Chaula SA, Tarimo DS. Impact of praziquantel mass drug administration campaign on prevalence and intensity of Schistosoma haemamtobium among schoolchildren in Bahi district, Tanzania. *Tanzan J Health Res*. 2014; 16:1–8
32. Ahmed KS, Siraj NM, Fitsumberhan H, Isaac S, Yohannes S, Eman D, *et al*. Knowledge, attitude and practice (KAP) assessment of intestinal parasitic infection among school children in Asmara. *Eritrea Health*. 2016; 9: 57. <https://doi.org/10.4236/HEALTH.2017.91005>
33. Kabatereine N, Fleming F, Thuo W, Tinkitina B, Tukahebwa EM, Fenwick A. Community perceptions, attitude, practices and treatment seeking behavior for schistosomiasis in Lake Victoria islands in Uganda. *BMC Res Notes*. 2014; 7:900. <https://doi.org/10.1186/1756-0500-7-900>
34. Macharia JW, Ng'ang'a ZW, Njenga SM. Factors influencing community participation in control and related operational research for urogenital schistosomiasis and soil-transmitted helminths in rural villages of Kwale County, coastal Kenya. *Pan Afr Med J*. 2016; 24:136. doi: 10.11604/pamj.2016.24.136.7878
35. Kaliyaperumal K. Guideline for conducting a knowledge, attitude and practice (KAP) study.  *AECS illumination.* 2004;4:7–9. Available at: <http://v2020eresource.org/content/files/guideline_kap_Jan_mar04.pdf> (Acessed on 12Sep2022)
