# Supplementary material for: Knowledge, attitude and risky practices on schistosomiasis in Ethiopia: A scoping review
Source: PLoS One. 2025 Nov 14;20(11):e0337085. doi: 10.1371/journal.pone.0337085 (PMC12617891; doi:10.1371/journal.pone.0337085)
Supplement: Attachment — Submitted filename: NOTE TO THE EDITOR.docx [file pone.0337085.s008.docx]

NOTE TO THE EDITOR

This manuscript is a review article and not the product of a primary research. Therefore does not fall within the scope of the journal and is recommended for rejection.
