# Supplementary material for: Knowledge, attitude and risky practices on schistosomiasis in Ethiopia: A scoping review
Source: PLoS One. 2025 Nov 14;20(11):e0337085. doi: 10.1371/journal.pone.0337085 (PMC12617891; doi:10.1371/journal.pone.0337085)
Supplement: Attachment — Submitted filename: Response to Reviewers.docx [file pone.0337085.s011.docx]

**Responses to Reviewers’ Comments and Questions**

Dear editor and reviewers, thank you for your constructive comments which all are important inputs for the betterment of the manuscript. Below we tried to respond to all the comments/questions one by one; we also have incorporated all the corrections in the revised manuscript (shown as highlighted).

***Editor comments***

1. Please ensure that your manuscript meets PLOS ONE's style requirements, including those for file naming.

**Response**: We have prepared and revised the manuscript based on the journal style requirements

2. We note that your Data Availability Statement is currently as follows: [All data are available in the manuscript]. Please confirm at this time whether or not your submission contains all raw data required to replicate the results of your study.

**Response**: we accept this comment. All extracted data are presented in Table 1 in the manuscript.

An excel data containing list of screened articles (after removing duplicates) and screening result (inclusion/exclusion) based on the title, abstract or full text, together with reasons for excluding studies, is submitted in supplementary file S1. In addition, a table containing name of data extractors, extraction date, and raw data extracted is submitted in Supplementary file S3. We have no other data available. Based on this, we have revised the Data availability statement in the revised submission.

3. We note that there is identifying data in the Supporting Information file <Quality assesment.docx>.

**Response:** To avoid identifying data, we have removed the author names and references, and we have coded included studies by letters (A to J).

4. Please review your reference list to ensure that it is complete and correct.

**Response**: We have reviewed the reference list to make it complete and correct. Fortunately, all our references are not retracted.

5. As required by our policy on Data Availability, please ensure your manuscript or supplementary information includes the following:
   A numbered table of all studies identified in the literature search, including those that were excluded from the analyses. For every excluded study, the table should list the reason(s) for exclusion.

**Response:** we have submitted this in an excel file as supplementary file S1

If any of the included studies are unpublished, include a link (URL) to the primary source or detailed information about how the content can be accessed.

**Response:** all included studies are published

A table of all data extracted from the primary research sources for the systematic review and/or meta-analysis. The table must include the following information for each study:
Name of data extractors and date of data extraction
Confirmation that the study was eligible to be included in the review.

All data extracted from each study for the reported systematic review and/or meta-analysis that would be needed to replicate your analyses.
**Response:** comment accepted and we have submitted the table containing all the requested contents as Supplementary file S3.

If data or supporting information were obtained from another source (e.g. correspondence with the author of the original research article), please provide the source of data and dates on which the data/information were obtained by your research group.
**Response:** No data or supporting information was obtained from other sources than the eligible reviewed studies.

If applicable for your analysis, a table showing the completed risk of bias and quality/certainty assessments for each study or outcome.  Please ensure this is provided for each domain or parameter assessed. For example, if you used the Cochrane risk-of-bias tool for randomized trials, provide answers to each of the signaling questions for each study. If you used GRADE to assess certainty of evidence, provide judgements about each of the quality of evidence factor. This should be provided for each outcome.

**Response:** we have submitted a table containing quality assessment results of each reviewed study in Supplementary file S2.
An explanation of how missing data were handled.

**Response:** not applicable for our review, because we didn’t do quantitative analysis for any outcome.
 **Reviewers' comments to the author:**

1. Is the manuscript technically sound, and do the data support the conclusions?
   The manuscript must describe a technically sound piece of scientific research with data that supports the conclusions. Experiments must have been conducted rigorously, with appropriate controls, replication, and sample sizes. The conclusions must be drawn appropriately based on the data presented.

Reviewer #1: Yes

Reviewer #2: Yes

Reviewer #3: Yes

Reviewer #4: Yes

Reviewer #5: Yes

Reviewer #6: Yes

2. Has the statistical analysis been performed appropriately and rigorously?

Reviewer #1: I Don't Know

Reviewer #2: No

Reviewer #3: I Don't Know

Reviewer #4: Yes

Reviewer #5: Yes

Reviewer #6: N/A

**Response**: We have summarized data only based on thematic analysis. As we explained in the ‘Limitations’ section, due to differences in KAP assessment tools across reviewed studies, we couldn’t do meta-analysis or regression to present pooled results.

3. Have the authors made all data underlying the findings in their manuscript fully available?
    Reviewer #1: Yes

Reviewer #2: No

Reviewer #3: Yes

Reviewer #4: Yes

Reviewer #5: Yes

Reviewer #6: Yes

**Response**: extracted data are presented in table 1 in the manuscript. Additional data (list of studies screened studies and their eligibility results, included studies quality assessment result, and extracted data) are submitted as supplementary file S1, S2 and S3.

4. Is the manuscript presented in an intelligible fashion and written in Standard English?
    Reviewer #1: Yes

Reviewer #2: Yes

Reviewer #3: Yes

Reviewer #4: Yes

Reviewer #5: Yes

Reviewer #6: Yes

**Response**: More language editions are made based on comments attached by reviewers 2 and 6.

5. **Review Comments to the Author**

**Reviewer #1:**

Introduction
Please recast here ‘’The disease is endemic in 70 developing countries, and more than 200
million people are infected worldwide’’.
**Response**: comment accepted and the sentence is rephrased.
Please recast here and don’t start with ‘’Both’’ Both the intestinal (caused by S. mansoni) and
urogenital (caused by S. haematobium) forms of SCH are prevalent in Ethiopia [4].
**Response**: comment accepted and the sentence is rephrased.
Please recast here and break into two sentences ‘’Mass drug administration (MDA), facility-based case diagnosis and treatment, snail control in hotspots, SBCC, and access to safe water supply, basic sanitation, and hygiene (WASH) are the five strategies identified to achieve this goal [4].’’
**Response**: We have made language editions but despite we understand it is a long sentence, we prefer to make it one sentence as it contains a list of equally valued interventions.

Please recast here ‘’Even though, a few studies were conducted in Ethiopia, the previous review did not include them with a possible reason of robust eligibility criteria and review period’’
**Response**: comment accepted and the sentence is rephrased in the revised submission

Discussion
Please recast here ‘’Apart from inadequate health education and promotion, socio-cultural factors might also be barriers to changes in attitude and practices towards SCH control and prevention [15].’’
**Response**: comment accepted and the sentence is rephrased in the revised submission

Please recast here ‘’According to this review, comprehensive knowledge about sources of infection, routes and modes of transmission, morbidity, treatment, and prevention of SCH is lacking in Ethiopia.’’
**Response**: comment accepted and the sentence is rephrased in the revised submission

Please recast here ‘’For instance, we observed that school and community-based education sessions
were conducted only at the time of deworming which is an inadequate method to establish good knowledge and change community behavior. ‘’
**Response**: comment accepted and the sentence is rephrased in the revised submission

Please recast here ‘’If it is properly implemented, health education has a high input-output
ratio and is a cost-effective prevention and control method’’.
**Response**: comment accepted and the sentence is rephrased in the revised submission

Please recast here ‘’Having comprehensive knowledge contributes to having a positive attitude, which in turn contributes to behavioral changes like reducing risky activities’’
**Response**: comment accepted and the sentence is rephrased in the revised submission

Please recast here ‘’Variations in the health education implementation at different transmission settings and study populations might contribute to differences in behavioral changes in Ethiopia.’’
**Response**: comment accepted and the sentence is rephrased in the revised submission

Conclusions
Please recast here ‘’Findings in the reviewed studies showed inadequate KAP level of people, despite both community and school-based health education platforms were established and have been implemented for more than a decade’’.

**Response**: comment accepted and the sentence is rephrased in the revised submission

**Reviewer #2**: General Reviewer Observations and Comments
A systematic review on the knowledge, attitude, and practice (KAP) regarding schistosomiasis in Ethiopia reveals several critical insights. Generally, knowledge about schistosomiasis is variable, often influenced by factors such as education, geographic location, and access to health information.
Many communities have limited understanding of the transmission dynamics, symptoms, and prevention strategies associated with the disease. This gap in knowledge frequently translates to negative attitudes towards preventive measures, with some cultural beliefs affecting perceptions of the disease.
Moreover, behavioral practices associated with schistosomiasis prevention, including safe water usage and sanitation, are often inadequate.
Lack of access to clean water and proper sanitation facilities exacerbates the situation, contributing to the persistence of the disease in endemic regions.
Comments for review
• There is a lot of systematic editing and reviewing required from the investigator.

**Response**: comment accepted and we have made intense language editions based on suggestions in the attached PDF file from reviewer 2 and MS word doc attached by reviewer 6 (all editions are highlighted).

• When conducting research or a systematic review on knowledge, attitude, and practice (KAP) regarding schistosomiasis, various statistical analysis methods and data presentation tools should be used which are missing in the Data Analaysis section:
• Gaps in statistical analysis methods and the team could have used one of the following:
o Descriptive Statistics: Mean, Median, Mode: To summarize central tendencies in knowledge scores or attitudes.
o Frequency and Percentage: To detail respondents' knowledge levels, attitudes, and practices concerning schistosomiasis.
o Inferential Statistics: Chi-Square Test: To examine associations between categorical variables (e.g., knowledge levels across different demographic groups).
o T-Tests or ANOVA: To compare means of knowledge or attitude scores between different groups, such as educational levels or regions.
o Correlation Analysis: To assess relationships between knowledge and practice scores using Pearson or Spearman correlation coefficients.
o Regression Analysis: To identify factors influencing practices regarding schistosomiasis (e.g., logistic regression for binary outcomes).
o Factor Analysis: To identify underlying relationships between different KAP components and determine key factors affecting knowledge and practices.
Gaps in Data Presentation/Graphs/Charts and the team could have used one of the following:
o Tables: For presenting descriptive statistics, frequency distributions, and results of inferential analyses clearly.
o Bar Charts: To represent categories of knowledge and attitudes visually.
o Pie Charts: For displaying proportions of respondents' attitudes or practices.
o Box Plots: To show distributions of knowledge scores across different groups.
o Geospatial Analysis Tools: If location data is available, tools like ArcGIS can visualize schistosomiasis prevalence and KAP data across different geographic areas.
Using these statistical methods and presentation tables would have enhanced the clarity and impact of the findings related to KAP on schistosomiasis in Ethiopia.

**Response:** Dear reviewer, we appreciate your detail explanation and suggestion regarding statistical analysis and we have understood your concern very well. However, we didn’t use statistical analysis in this review because reviewed studies included varied study population (school-aged children, community, mothers of pre-school-aged children), and tools used to measure KAP varied across reviewed studies. Therefore, generating pooled results using meta-analysis or other statistical analyses is impossible. Rather a thematic analysis was used to present summarized results of reviewed studies. We have mentioned this in the ‘limitations’ section of the manuscript. Please check that similar reviews were published following the same method as ours (<https://pubmed.ncbi.nlm.nih.gov/29347919/>)

**Reviewer #3**: Dear Author, thank you for submitting your interesting review. I think the data reported are of great interest and presented in an adequate fashion. Written English does not need corrections. Discussion and Introduction are appropriate.

**Response**: dear reviewer, thank you for reviewing our manuscript, and for giving credit for our work

**Reviewer #4**: I hereby recommend the manuscript to be published in the Plos One journal. The article on the systematic review of "Knowledge, attitude and practice on schistosomiasis in Ethiopia" has been extensively analyzed based on the studies on the disease between 2006 and 2023 in Ethiopia. Although the analysis revealed inadequate KAP on the disease but the publication of this research will further stress the need for the execution of health education and constant surveillance of schistosomiasis in Ethiopia for sustained prevention and control of the disease.

**Response**: dear reviewer, thank you for reviewing our manuscript, and recommendation for publication in PLoS One.

**Reviewer #5**: Important work
Complements the previous information.
Findings in the reviewed studies showed inadequate KAP level of people, despite both community and school-based health education platforms were established and have been implemented for more than a decade. This reminds for future strengthening the health education program and monitoring the practical implementation and impact assessment of the existing programs. Also, the implementation of two programs together (namely, STH and SCH), should be precise enough to avoid confusion and mixed information. Preparing a standard context-specific KAP assessment tool could be an immediate responsibility of the FMoH NTD prevention and control program. More studies are also recommended both at school and community level to explore the KAP level and associated factors at different endemicity settings among all at-risk population groups.

**Response**: dear reviewer, thank you for reviewing our manuscript

**Reviewer #6**: The authors need to clarify a statement where four studies were referred but five results were presented under the section on knowledge about schistosomiasis from the papers reviewed. A comment on this was inserted. The language style was for the most part very passive. Although this is a review article, it is advised that some of the style be revised to be more active, a few of this has been addressed.

**Response for comments from reviewer 6**:

A. Dear reviewer, please accept our apology for the write up error under the section “knowledge about schistosomiasis”. The result ‘1.7% [25]’ was wrongly included and we have removed it in the revised manuscript. To avoid such other mistakes, we have double checked all the results from the extracted data table and from reviewed papers.
B. We have accepted and incorporated all grammar corrections given by track change in the attached manuscript from you (reviewer 6).
C. To avoid all passive language, we have rephrased sentences (all changes are highlighted)
D. You (reviewer 6) have written a ‘NOTE TO THE EDITOR’ which says “This manuscript is a review article and not the product of a primary research. Therefore does not fall within the scope of the journal and is recommended for rejection”. We politely disagree with your justification because PLOS ONE publishes systematic reviews. Please visit <https://journals.plos.org/plosone/s/journal-information#loc-scope>
