# Supplementary material for: Knowledge, attitude and risky practices on schistosomiasis in Ethiopia: A scoping review
Source: PLoS One. 2025 Nov 14;20(11):e0337085. doi: 10.1371/journal.pone.0337085 (PMC12617891; doi:10.1371/journal.pone.0337085)
Supplement: Attachment — Submitted filename: Plos one Review December.docx [file pone.0337085.s010.docx]

**Plos one Review**

**Title: Knowledge, attitude and practice on schistosomiasis in Ethiopia. A systematic review**

I am not convinced the selected studies were illegible for a systematic review.

Is this a scoping review or a systematic review?

-The authors lack knowledge of the difference between scoping review and systematic review

May I request that the authors decide if they want to do a scoping or systematic review using the following table?

| **Feature** | **Systematic Review** | **Scoping Review** |
| --- | --- | --- |
| **Purpose** | Answer a specific, focused research question | Map the breadth and scope of research |
| **Research Question** | Specific, focused, often hypothesis-driven | Broad, exploratory |
| **Methodology** | Structured, with critical appraisal and synthesis | Flexible, with categorisation and mapping |
| **Data Analysis** | Quantitative synthesis (e.g., meta-analysis) | Qualitative summary, no pooling of data |
| **Outcome** | Evidence-based conclusion or recommendation | Overview of research landscape, gaps, and trends |
| **Usefulness** | Decision-making for practice or policy | Identifying research gaps, informing future reviews |

**Abstract**

**Background: the first sentence is not specific to the main focus of the review, which is** **schistosomiasis**

Methods:

- which framework was used to guide the systematic review?
- What search strategy was used to search literature in electronic databases
- Which databases were selected and why?
- What was the duration of the search
- What was used to document the selection process

Results: the results of the review are presented quantitatively

- It is also a requirement that authors mention the type of studies they found through the search

What are the strengths and limitations of this systematic review?

Methodology: Major revision is required

- A **systematic review** is a comprehensive, structured approach to synthesizing research evidence on a specific topic or question. It follows a clear, predefined methodology to minimize bias, ensuring the review is transparent, reproducible, and reliable. The current methodology is not articulated clearly. The following items are not fully described to a level where the manuscript can be accepted and published by a journal,

- There is no specific, clear, answerable research question. This typically follows the **PICO** framework (Population, Intervention, Comparison, Outcome) for clinical questions or other relevant frameworks for different fields.

**According to the Authors,** this review aimed to evaluate the KAP concerning SCH among different population groups in Ethiopia; this aim is written in the background. Another aim written in the methodology reads as follows: We aimed to assess KAP-level progress towards SCH in Ethiopia after the launch of the Health Extension Program

(2004–2005) what is the exact aim/ research question of this review

**Registration**: Many systematic reviews are registered with platforms like **PROSPERO** (for health-related reviews) to prevent duplication and ensure transparency; it's unclear if this review has been registered to avoid duplication

**Comprehensive Literature Search**

- **Database Selection**: Reviews require Searching multiple relevant databases (e.g., PubMed, Scopus, Web of Science, Cochrane Library) to find studies related to the research question. Please revise accordingly.
- **Search Strategy**: The authors used Boolean only. What about medical subject headings (MeSH)?
- **Gray Literature**: the authors should minimise publication bias and consider including non-peer-reviewed sources like theses, conference proceedings, and government reports.

**Screen and Select Studies**

Title and Abstract Screening: what were the inclusion and exclusion criteria for the review?

Full-text Screening: what were the eligibility criteria for the review?

**Quality assessment versus Assessment of Risk of Bias**

Normally, systematic reviews use an assessment of Risk of Bias: Evaluate the quality of studies using Cochrane's Risk of Bias tool to assess methodological rigour. I haven't seen this assessment.

It is common to have two independent reviewers screen studies to reduce bias. Were there any disagreements which required consensus or a third reviewer?

**Assessment of the Study Quality**

- **Risk of Bias**: how did the authors Evaluate the quality and risk of bias in the included studies, and which standardized tools did they use (e.g., Cochrane Risk of Bias tool, ROBINS-I for non-randomized studies)?
- **Critical Appraisal**: how did the authors appraise the included studies? Consider factors like study design, sample size, methodology, and funding sources that may influence the validity of the findings.

**. Synthesize the Data: Synthesis is insufficient due to the limited number of studies that have included data.**

- **Quantitative Synthesis (Meta-analysis)**: where the studies are not sufficiently homogeneous (in terms of design, participants, interventions, and outcomes), conduct a meta-analysis to combine the results quantitatively.
  - Calculate effect sizes (e.g., risk ratios, mean differences) and assess study heterogeneity. The authors haven’t done this.
- **Qualitative Synthesis**: If the studies are too heterogeneous for a meta-analysis, provide a narrative synthesis, summarizing key findings across studies, highlighting patterns, and identifying gaps in evidence.
- **Sensitivity Analyses**: Conduct sensitivity analyses to assess how robust the findings are to changes in the analysis, such as excluding studies with a high risk of bias.

**Assess the Quality of Evidence (GRADE)**

As per the previous comments from the reviewers, the authors haven’t applied this**.**
