# Supplementary material for: Knowledge, attitude and risky practices on schistosomiasis in Ethiopia: A scoping review
Source: PLoS One. 2025 Nov 14;20(11):e0337085. doi: 10.1371/journal.pone.0337085 (PMC12617891; doi:10.1371/journal.pone.0337085)
Supplement: Attachment — Submitted filename: Response to Reviewers R2.docx [file pone.0337085.s012.docx]

**Responses to Reviewers’ Comments and Questions**

Dear editor and reviewers, thank you for your action and comments based on our revised manuscript.

Below we tried to respond to all the comments/questions one by one; we also have incorporated all the corrections in the revised manuscript (shown as highlighted).

**Comments to the Author**
**Reviewer #2**:

Generally, knowledge about schistosomiasis is variable, often influenced by factors such as education, geographic location, and access to health information. Many communities have a limited understanding of the transmission dynamics, symptoms, and prevention strategies associated with the disease. This gap in knowledge frequently translates to negative attitudes towards preventive measures, with some cultural beliefs affecting perceptions of the disease. Moreover, behavioral practices associated with schistosomiasis prevention, including safe water usage and sanitation, are often inadequate. Lack of access to clean water and proper sanitation facilities exacerbates the situation, contributing to the persistence of the disease in endemic regions.
**Response:** dear reviewer, we totally accept your impression and we also believe knowledge, attitudes and preventive practices are closely inter-related.
**Comments for review**
There is a lot of systematic editing and reviewing required from the investigator. When conducting research or a systematic review on knowledge, attitude, and practice (KAP) regarding schistosomiasis, various statistical analysis methods and data presentation tools should be used which are missing in the Data Analysis section
Gaps in statistical analysis methods and the team could have used one of the following:
o Descriptive Statistics: Mean, Median, Mode: To summarize central tendencies in knowledge scores or attitudes.
o Frequency and Percentage: To detail respondents' knowledge levels, attitudes, and practices concerning schistosomiasis.
o Inferential Statistics: Chi-Square Test: To examine associations between categorical variables (e.g., knowledge levels across different demographic groups).
o T-Tests or ANOVA: To compare means of knowledge or attitude scores between different groups, such as educational levels or regions.
oCorrelation Analysis: To assess relationships between knowledge and practice scores using Pearson or Spearman correlation coefficients.
oRegression Analysis: To identify factors influencing practices regarding schistosomiasis (e.g., logistic regression for binary outcomes).
oFactor Analysis: To identify underlying relationships between different KAP components and determine key factors affecting knowledge and practices.
Gaps in Data Presentation/Graphs/Charts and the team could have used one of the following:
oTables: For presenting descriptive statistics, frequency distributions, and results of inferential analyses clearly.
oBar Charts: To represent categories of knowledge and attitudes visually.
oPie Charts: For displaying proportions of respondents' attitudes or practices.
oBox Plots: To show distributions of knowledge scores across different groups.
oGeospatial Analysis Tools: If location data is available, tools like ArcGIS can visualize schistosomiasis prevalence and KAP data across different geographic areas.
Using these statistical methods and presentation tables would have enhanced the clarity and impact of the findings related to KAP on schistosomiasis in Ethiopia.

**Response:** Dear reviewer**,** based on other reviewers’ and our own later evaluation of the review, we agreed that the review we conducted should be “ a scoping review” rather than “ a systematic review” and we have made this correction to the revised manuscript. Because, reviewed studies included varied study population (school-aged children, community, mothers of pre-school-aged children), and tools used to measure KAP varied across reviewed studies; i.e. not focused to a specific question targeting a certain study group, and tools used in included studies varied. Therefore, generating pooled results using meta-analysis or other statistical analyses, which are requirements of a systematic review. Rather a thematic analysis was used to present summarized results of reviewed studies. We have mentioned this in the ‘limitations’ section of the manuscript. Thematic analysis is a feature of thematic or scoping review.

Reviewer #7: Dear Editor,
Thank you very much for sending me the revised article titled " Knowledge, attitude and practice on schistosomiasis in Ethiopia. A systematic review " for re-review.
I would like to inform you that in my opinion, the author has responded to all the comments in the revised article and this article is worthy of publication in a valuable journal.

**Response**: Thank you for your constructive comments

Reviewer #8: (No Response)

**Attachment comment**

**Plos one Review**

**Title: Knowledge, attitude and practice on schistosomiasis in Ethiopia. A systematic review**

I am not convinced the selected studies were illegible for a systematic review.

Is this a scoping review or a systematic review?

-The authors lack knowledge of the difference between scoping review and systematic review

May I request that the authors decide if they want to do a scoping or systematic review using the following table?

| **Feature** | **Systematic Review** | **Scoping Review** |
| --- | --- | --- |
| **Purpose** | Answer a specific, focused research question | Map the breadth and scope of research |
| **Research Question** | Specific, focused, often hypothesis-driven | Broad, exploratory |
| **Methodology** | Structured, with critical appraisal and synthesis | Flexible, with categorisation and mapping |
| **Data Analysis** | Quantitative synthesis (e.g., meta-analysis) | Qualitative summary, no pooling of data |
| **Outcome** | Evidence-based conclusion or recommendation | Overview of research landscape, gaps, and trends |
| **Usefulness** | Decision-making for practice or policy | Identifying research gaps, informing future reviews |

**Response**: Thank you for the comment with detailed explanation. Based on your explanation and discussion among authors, we authors agreed that our review is “a scoping review” rather than “a systematic review”. We have made this correction to the revised submission. Despite we followed PRISMA guideline to search and review literatures, the data we extracted couldn’t be analysed in a way required for systematic reviews. Because, reviewed studies varied by study population (school-aged children, community, mothers of pre-school-aged children), and tools used to measure KAP varied across reviewed studies; i.e. not focused to a specific question targeting a certain study group, and tools used in included studies varied.

**Abstract**

Background: the first sentence is not specific to the main focus of the review, which is Schistosomiasis

**Response**: we accept this comment and we have revised the sentence (highlighted)

Methods:

- which framework was used to guide the systematic review?
- What search strategy was used to search literature in electronic databases
- Which databases were selected and why?
- What was the duration of the search
- What was used to document the selection process

**Response:** We have included all the above queries in the ‘methods’ section of the abstract in the revised manuscript. But we didn’t justify why we use the mentioned data bases just to avoid lengthy abstract. For your information, we used those mentioned electronic search databases because those are the only data bases we accessed freely.

Results: the results of the review are presented quantitatively

- It is also a requirement that authors mention the type of studies they found through the search

**Response**: all were cross-sectional studies (mentioned in the revised manuscript). This is also presented in ‘the type of study’ column in table 1.

What are the strengths and limitations of this systematic review?

**Response**: presented in the ‘strengths and limitations of the review’ section.

Methodology: Major revision is required

- A **systematic review** is a comprehensive, structured approach to synthesizing research evidence on a specific topic or question. It follows a clear, predefined methodology to minimize bias, ensuring the review is transparent, reproducible, and reliable. The current methodology is not articulated clearly. The following items are not fully described to a level where the manuscript can be accepted and published by a journal,

- There is no specific, clear, answerable research question. This typically follows the **PICO** framework (Population, Intervention, Comparison, Outcome) for clinical questions or other relevant frameworks for different fields.

**According to the Authors,** this review aimed to evaluate the KAP concerning SCH among different population groups in Ethiopia; this aim is written in the background. Another aim written in the methodology reads as follows: We aimed to assess KAP-level progress towards SCH in Ethiopia after the launch of the Health Extension Program

(2004–2005) what is the exact aim/ research question of this review

**Response**: The aim of the review was to evaluate the KAP concerning SCH among different population groups in Ethiopia. In the methodology, the phrase “ to assess KAP-level progress towards SCH in Ethiopia after the launch of the Health Extension Program” was put to explain why we include studies published only after 2005 (start of the health extension program); we have rephrased this to make it clear.

**Registration**: Many systematic reviews are registered with platforms like **PROSPERO** (for health-related reviews) to prevent duplication and ensure transparency; it's unclear if this review has been registered to avoid duplication

**Response**: we didn’t register the review in any such platform

**Comprehensive Literature Search**

- **Database Selection**: Reviews require Searching multiple relevant databases (e.g., PubMed, Scopus, Web of Science, Cochrane Library) to find studies related to the research question. Please revise accordingly.
- **Search Strategy**: The authors used Boolean only. What about medical subject headings (MeSH)?
- **Gray Literature**: the authors should minimise publication bias and consider including non-peer-reviewed sources like theses, conference proceedings, and government reports.

**Response**: Regarding databases, we didn’t search in scopus, web of science and cocrane because we have no access to those databases. We have made gray literatures but we didn’t get any unpublished eligible literatures.

**Screen and Select Studies**

Title and Abstract Screening: what were the inclusion and exclusion criteria for the review?

Full-text Screening: what were the eligibility criteria for the review?

**Quality assessment versus Assessment of Risk of Bias**

Normally, systematic reviews use an assessment of Risk of Bias: Evaluate the quality of studies using Cochrane's Risk of Bias tool to assess methodological rigour. I haven't seen this assessment.

It is common to have two independent reviewers screen studies to reduce bias. Were there any disagreements which required consensus or a third reviewer?

**Assessment of the Study Quality**

- **Risk of Bias**: how did the authors Evaluate the quality and risk of bias in the included studies, and which standardized tools did they use (e.g., Cochrane Risk of Bias tool, ROBINS-I for non-randomized studies)?
- **Critical Appraisal**: how did the authors appraise the included studies? Consider factors like study design, sample size, methodology, and funding sources that may influence the validity of the findings.

**. Synthesize the Data: Synthesis is insufficient due to the limited number of studies that have included data.**

- **Quantitative Synthesis (Meta-analysis)**: where the studies are not sufficiently homogeneous (in terms of design, participants, interventions, and outcomes), conduct a meta-analysis to combine the results quantitatively.
  - Calculate effect sizes (e.g., risk ratios, mean differences) and assess study heterogeneity. The authors haven’t done this.
- **Qualitative Synthesis**: If the studies are too heterogeneous for a meta-analysis, provide a narrative synthesis, summarizing key findings across studies, highlighting patterns, and identifying gaps in evidence.
- **Sensitivity Analyses**: Conduct sensitivity analyses to assess how robust the findings are to changes in the analysis, such as excluding studies with a high risk of bias.

**Assess the Quality of Evidence (GRADE)**

As per the previous comments from the reviewers, the authors haven’t applied this**.**

**Response:** Overall, the nature of the literature we reviewed are not fully eligible to perform all the comments you provided here. That is why we revised the review to be a scoping review. So, we couldn’t address all your comments regarding data quality and analysis
