# Supplementary material for: Knowledge, attitude and risky practices on schistosomiasis in Ethiopia: A scoping review
Source: PLoS One. 2025 Nov 14;20(11):e0337085. doi: 10.1371/journal.pone.0337085 (PMC12617891; doi:10.1371/journal.pone.0337085)
Supplement: Attachment — Submitted filename: Response to Reviewers R4.docx [file pone.0337085.s016.docx]

**Responses to Reviewers’ Comments and Questions**

Dear editor and reviewers, thank you for your action and comments based on our revised manuscript.

Below we tried to respond to all the comments/questions one by one; we also have incorporated all the corrections in the revised manuscript (shown as highlighted).

**Reviewer #7:** Accept
all required questions have been answered and that all responses meet formatting specifications.

**Reflection**: Thank you for your positive response

**Reviewer #9:** Generally, your document has merit but needs major revision. your need to address all the issues raised in your main document. are you interested in KAP about the disease of its prevention and control practices? so, you need to clarify your title. it is on prevention, about SCH, etc.??? you need to clearly specify it. the document has many typo errors and needs major edition.

**Response**: We accept the comment and have made revision on the title. We were interested in knowledge and attitudes towards SCH, as well as, risky activities practiced by the community (not on prevention). We have made tense and grammar corrections throughout the document (all are highlighted).
