# Supplementary material for: Knowledge, attitude and risky practices on schistosomiasis in Ethiopia: A scoping review
Source: PLoS One. 2025 Nov 14;20(11):e0337085. doi: 10.1371/journal.pone.0337085 (PMC12617891; doi:10.1371/journal.pone.0337085)
Supplement: Attachment — Submitted filename: Response to Reviewers R5.docx [file pone.0337085.s017.docx]

**Responses to Reviewers’ Comments and Questions**

Dear editor and reviewers, thank you for your action and comments based on our revised manuscript.

Below we tried to respond to all the comments/questions one by one; we also have incorporated all the corrections in the revised manuscript (shown as highlighted).

**Reviewer #7:**

Dear editor, many thanks for your kind review invitation a manuscript " Knowledge, attitude and risky practice on schistosomiasis in Ethiopia: A scoping review ". I would like to inform you that I had accepted the manuscript in revision-3 style.

**Response**: thank you for accepting the manuscript

**Reviewer #9:**

Overall, the manuscript has a merit. To make the manuscript sound for readers, it is better if you consider my comments that I forwarded in the main manuscript.

**Response**: We accepted all the comments given in the main manuscript and have made revisions accordingly (all changes are highlighted in the revised submission).

**Comments not accepted in the abstract**

1. Do not use abbreviations in the abstract

**Reason**: It is not recommended to use abbreviations in the abstract; there is no hard rule that totally prohibit use of abbreviations in the abstract. Hence, we use a few abbreviations just to avoid lengthy abstract.

2. Try to include the discriptive statistcis used to report the result.

**Reason**: we just synthesized and presented the summarized data. We didn’t do any statistical analysis.

3. It is better if you report what you found. This is not summary. You should summarize the result you found. like 29% had poor attitude etc.

**Reason**: Reviewed studies used quite varied KAP assessment tools and the reporting was not consistent. So, it is difficult to pool results. Rather we presented main findings of each study separately.

**In the study selection**

**Question**: why did you exclude articles before 2006?

**Response**: Health education and promotion on SCH started in 2004 when the health extension program became operational in Ethiopia. So, we aim to assess the progress in KAP after the start of the health education program.

**Questions in the results section**

Previously. I have asked you to show whether this articles were retreived or not. do you think all this are removed because of duplicates?

**Response**: these articles were retrieved and all these were removed because they were duplicates. We used the less specific ‘ google scholar’ which makes large number of duplicates.

**Reviewer #10:**

1. Title and Abstract
The phrase “risky practice” is grammatically incorrect; it should be “risky practices”.

**Response**: we accept the comment revised accordingly
The abstract is clear in stating the background and purpose. However, quantitative data should be provided where possible (e.g., number of studies included, participant characteristics).

**Response**: We accept the comment and revised accordingly
Avoid vague statements like “studies found large differences…”—instead, provide specific findings or percentages for clarity.

**Response**: We accept the comment but because each study used different tools and inconsistent reporting, it is difficult to put pooled quantitative results.

2. Introduction
Clearly define what is meant by “risky practices” early on in this section. Is this referring to water contact, open defecation, etc.?

**Response**: yes, it is referring to practices that predispose for *Schistosoma* infection (water contact, open defication…)

3. Methods
The search strategy is appropriate but needs more transparency. Provide the full search string, date last searched, and whether the search was peer-reviewed (PRESS standard).

**Response**: We accept the comment and revised accordingly
Clarify why Google Scholar was included given its lower specificity for peer-reviewed literature.

**Response**: we agree that google scholar is less specific but we used it for literature search jus not to miss studies to be included.
4. Results
How were “good” or “poor” knowledge/attitudes/practices defined across studies? This lack of standardization complicates synthesis.

**Response**: Yes, there was inconsistency in defining good or poor KAP. We have explained this limitation (lack of standard cut off values) in the last paragraph of the ‘discussion’ section.
