# Supplementary material for: Knowledge, attitude and risky practices on schistosomiasis in Ethiopia: A scoping review
Source: PLoS One. 2025 Nov 14;20(11):e0337085. doi: 10.1371/journal.pone.0337085 (PMC12617891; doi:10.1371/journal.pone.0337085)
Supplement: Attachment — Submitted filename: Reviewer Comments.pdf [file pone.0337085.s005.pdf]

Reviewer Comments

Title: Knowledge, attitude and practice on schistosomiasis in Ethiopia. A systematic review
Manuscript Ref: PONE-D-24-22605
Article Type: Research Article

Abstract

Background: Health education and promotion enable community involvement and active participation in disease control and prevention. Therefore, it is considered as one of the key strategies to prevent and control schistosomiasis (SCH) in Ethiopia. However, comprehensive data on the knowledge, attitude and practice (KAP) of vulnerable populations towards the disease are lacking. Methods: A systematic search for studies published in Ethiopia between 2006 and 2023 was conducted in PubMed, Hinari, African Journal Online and Google Scholar using the keywords: "Schistosomiasis, Schistosoma, Schistosoma mansoni, Schistosoma haematobium, knowledge, attitude, Practice, Consciousness, Perception, Belief, Ethiopia" by combining with Bolean operators (AND, OR). Eligible studies were selected and reviewed according to the PRISMA guidelines and checklist. Thematic analysis was used to synthesize the selected studies.

Results: Knowledge gaps on the source of Schistosoma infection, transmission, morbidity, treatment and prevention in Ethiopia were identified. Studies have found large differences in attitudes toward SCH in terms of the population at risk, the severity of the disease, and beliefs in the availability and success of its treatment and prevention. Furthermore, in most studies included in this review, the majority of participants had negative attitudes toward SCH. The majority of participants also engaged in risky water-related practices, which facilitated the ongoing transmission of SCH. KAP scores among community members, school-aged children, and mothers/caregivers of preschool-aged children showed no significant differences.

Conclusions: The results of this systematic review showed that the KAP is inadequate despite health education platforms that have been established and implemented for many years. Therefore, we recommend strengthening the implementation of health education and continuous monitoring of SCH prevention and control activities.

Background

Schistosomiasis (SCH) is one of the 20 neglected tropical diseases (NTDs) and is caused by trematodes of the genus Schistosoma [1]. The disease is endemic in 70 developing countries and more than 200 million people are infected worldwide. *Schistosoma mansoni* and *S. haematobium* are the most widespread species both worldwide and in Africa. Sub-Saharan Africa (SSA) has the highest burden of SCH cases (90% of infections), and up to 20 million people suffer from severe chronic health consequences of the disease. The disability-adjusted life years lost due to SCH are estimated at 3.3 million [2]. Ethiopia is one of the countries in SSA with the highest burden of NTDs, including SCH [3]. A total of 966 districts were mapped between 2015 and 2020. Of these, 480 were endemic for SCH, and approximately 53.3 million people were at risk of infection. Both the intestinal (caused by *S. mansoni*) and urogenital (caused by *S. haematobium*) forms of SCH are common in Ethiopia [4]. Aware of this fact, the Federal Ministry of Health (BMG) launched the first NTD control program and implemented it between 2013 and 2015 [5]. During this period,

81.3% (10 million of 12.3 million) of affected school children (SAC) were dewormed with praziquantel. However, major challenges to sustaining interventions include lack of adequate attention to social behavior change communication (SBCC), poor supply of clean water, and low levels of sanitation and hygiene practices. The second national NTD strategic plan, which aimed to eliminate SCH to the point where it no longer poses a public health problem by 2020, was implemented from 2016 to 2020. Although 27 million SAC were treated during this period, SCH still represents a public health problem.

Currently, Ethiopia is implementing the 3rd National NTD Strategic Plan (2021-2025), which aims to eliminate Schistosoma transmission in Ethiopia by 2025. Mass drug administration (MDA), facility-based case diagnosis and treatment, snail control in hotspots, SBCC and access to safe water, basic sanitation and hygiene (WASH) are the five identified strategies to achieve this goal [4]. In addition to the national NTD strategic plans, the Health Extension Program, which began operations in Ethiopia in 2004–2005 and is currently being implemented, aimed to engage the community in disease prevention through the transfer of health knowledge and skills to households [6]. . However, preventable diseases such as SCH, soil-transmitted helminths (STHs), and malaria continue to pose major public health problems [4 , 7]. Finally, several studies have shown that reinfection after MDA is the main reason for persistent Schistosoma spp.

Transmission in many endemic areas [4, 8, 9]. Therefore, MDA should be implemented in integration with SBCC, WASH and vector control programs. Although SAC, women and other people whose occupations involve contact with natural waters are at higher risk of Schistosoma spp. are exposed. Infection [10, 11], the intervention programs mainly target SAC. Schistosomiasis prevention targeted at all vulnerable populations would have enabled successful control of the disease [12]. Successful implementation of SCH control and prevention strategies requires commitment and active community participation. Therefore, health education should ideally occur before programs that require full community involvement, as this will enable the community to make informed decisions about their participation [13]. In fact, aware of this fact, the World Health Organization (WHO) proposed as early as 1985 that health education and health promotion be the focus of SCH prevention work [14]. However, a systematic review of articles published in SSA between 2006 and 2016 found that the KAP value varied between studies [15]. Although some studies were conducted in Ethiopia, they were not included in the previous review, which may be due to strict eligibility criteria and review period. Health education was considered as one of the key strategies to control and eliminate SCH in previous Ethiopian national NTD strategic plans. However, changes in the population's KAP towards SCH have not been sufficiently taken into account. Even the existing information is fragmentary and there is no updated comprehensive national data in this regard. Therefore, the aim of this review was to evaluate the KAP in relation to SCH among different populations in Ethiopia. This systematic review will help stakeholders assess the impact of ongoing health education programs.

Methods

Search Strategy

We conducted a search for articles published between January 2006 and December 2023. Our aim was to assess KAP level progress towards SCH in Ethiopia after the launch of the Health

Extension Program (2004–2005). Two independent reviewers (GA and EN) searched literature in the databases Pubmed, Hinari, African Journal Online and Google Scholar using the keywords "*Schistosomiasis, Schistosoma, Schistosoma mansoni, Schistosoma haematobium,* knowledge, attitude, practice, awareness, perception, belief, Ethiopia ." The search was carried out by starting with broad terms, then narrowing them down step by step and combining keywords with Bolean operators (AND, OR). Additional research was conducted using the references listed in the identified publications. We conducted and reported this review in accordance with the Preferred Reporting Items for Systematic Reviews and Meta-Analyses (PRISMA) guidelines (Fig. 1).

Study Selection

Study selection was performed through a three-stage review process: database search, review of abstracts by two authors, and eligibility review, as shown in the PRISMA flowchart. The present study included studies conducted in Ethiopia between 2006 and 2023 that focused on a population's KAP to intestinal or urogenital schistosomiasis and published in English. Studies published before 2006 were excluded. Selected articles were carefully read to find the best available data to achieve the objective of this review.

Quality assessment

A quality assessment of the included studies was carried out using a method developed by Downes Brennan et al. adopted critical assessment tool for evaluating cross-sectional studies. [16]. The studies were assessed based on the following indicators: (1) clear definition of objectives; (2) study design consistent with stated objectives; (3) sample size justified; (4) target population clearly defined (appropriate population base/unbiased sample); (5) correctly measured risk factor and outcome variables using instruments that have been previously tested, piloted, or published; (6) methods (including statistical methods) sufficiently described to permit replication; (7) results for the analysis described in the methods presented; (8) Author discussions and conclusions supported by results; (9) limitations of the study discussed; (10) ethical approval or assent of participants. Responses for each indicator were scored 0 for "no" and 1 for "yes." The quality of papers was classified as low, medium and high if the total score was between 1-4, 5-7 and 8-10, respectively (Supplementary file 1). Accordingly, six and four studies were of medium and high quality, respectively.

Data extraction

We extracted data about the first author, year of publication, study aim, study design, study population, study location and key study results in a table. Thematic analysis was used for the overall synthesis of the selected studies.

Results

A total of 1310 articles were retrieved from all searched databases and websites. 644 articles were removed because they were duplicates. An additional 650 articles were removed after reading the titles and abstracts for the following reasons: 1) irrelevant to the title of the review (582); 2) conducted outside Ethiopia (64); 3) Reviews (3); 4) carried out before 2006 (2). An additional six were excluded because we were unable to retrieve full texts. Therefore, 10 articles

were included in the present review. After a detailed reading of each included study, four themes were identified: 1) sociodemographic characteristics; 2) knowledge about SCH; 3) attitude towards SCH; 4) Practices related to the prevention and control of SCH. Seven studies were community-based cross-sectional studies [17–23]; One study was conducted by SAC [24], while the remaining two studies recruited women with preschool-aged children (PSAC) [25, 26]. Three studies were conducted before the launch of the national school-based MDA in 2015 [17, 18, 25], while data for the remaining seven studies were collected after the launch of the MDA [19–24, 26]. The studies included in the present review recruited 4,763 participants (2,118 men and 2,645 women) aged between 5 and 86 years [17–20, 22–26], although one study did not report a maximum age limit [21]. Data on KAP related to SCH were collected using a structured questionnaire, except for one study in which a focus group discussion was conducted [21]. The ten studies reviewed are summarized in Table 1.

Knowledge about schistosomiasis

Six of the ten studies reported on the proportion of respondents who had heard of the disease SCH/schistosomiasis. In four articles, the majority of respondents: 87.2% [21], 80.8% [26], 71.5% [17] and 51.6% [24] had never heard of SCH. Two studies reported high levels of awareness among participants: 97% [19] and 69% [23] had heard of the disease. Three studies reported sources of information, with healthcare facilities/health professionals, family/friends and schools being the most important for most participants [19, 21, 26]. In most studies, there was little knowledge about the source and type of infection, and the majority of respondents answered “I don't know” when asked about both topics [17-20, 25, 26]: Related to the Source of Three studies reported good knowledge of SCH infection as the majority of respondents mentioned contaminated water [17, 21, 26], while three others reported poor knowledge [18, 23, 25]. Modes of SCH transmission were adequately reported in only one study, in which 88.6% (31/35) of participants responded that they were exposed to infection while swimming or bathing in contaminated water [21]. Of the participants who were aware of SCH, 28.9% [19], 50.4% [17], 60.4% [21], 79% [20] and 94% [26] did not know at least one sign or Symptom of SCH. In addition, most participants who knew the signs and symptoms were unable to comprehensively list the most common signs and symptoms. Respondents were asked: “Is schistosomiasis treatable?” in four studies. The proportion of participants who answered correctly was 1.7% [25], 21% [20], 29% [21], 78% [17] and 97.3% [19] among participants who ever from SCH had heard. In three studies, the majority of respondents - 66.7% [17], 79.6% [18] and 72.1% [19] - knew that SCH is preventable, while the other three studies reported poor knowledge, as 79 % [20] 87.2% [21] and 94% [26] of respondents did not know whether SCH can be prevented or not.

Attitude towards schistosomiasis

A significant number of respondents believed that drinking dirty water, consuming contaminated food, air, infected mosquitoes and flies are sources of Schistosoma infection [17–19, 24, 25]. In one study, 4 (1.2%) participants believed that contaminated food was a source of infection and 2 (0.6%) believed that cooking vegetables and hygiene practices could prevent SCH [18]. Another study reported that participants believed that eating contaminated food (23.7%, 145), dirty hands (16.2%, 99) and playing with soil (20%, 122) made them one Schistosoma infection predispose [24]. In one of the articles, 85% of respondents believed that intestinal SCH could not be transmitted by swimming or bathing in the river, crossing the river barefoot, washing clothes

in the river, or fishing in the river [26]. In another study, only 22.9% (11/48) of respondents agreed that there was a risk of Schistosoma infection from their contact with river or stream water. In the same study, the majority of 93.7% (45/48) reported that they were not sure whether SCH was a serious illness or not [21]. However, in two studies, 96% (289/301) [19] and 94% [22] of participants understood SCH as a serious illness. In the context of treatment and preventive chemotherapy, in one study, 67% (73/109) of participants agreed on the importance of MDA, but only 2.8% (3/109) agreed that mass therapy was effective for SCH control [21]; In another study, approximately 99.7% agreed on the importance of medication during SCH [19]. Finally, the majority of 94% [19] and 66.7% [17] of participants who were aware agreed that SCH is preventable, while 89.6% [21] and 79% [20] in others Studies did not agree with this fact. Risky Practices in Schistosoma Infection

Many of the participants had frequent contact with fresh water while swimming or bathing, washing clothes and utensils, crossing rivers barefoot, or fetching fresh water for household use [17-19, 21, 23, 24]. In two studies, 98% [18] and 56.5% [19] of participants who had heard the awareness frequently defecated in open fields and near rivers. In one study, 87.5% of respondents reported having a latrine, but rivers and stream banks in the study area were full of human waste during field observations [21]. Furthermore, in one study, 89.3% reported that they had never received health education on SCH prevention [18].

Discussion

This systematic review assessed knowledge, attitudes, and practices toward SCH among community members, SAC, and mothers/caregivers of PSAC in Ethiopia. Although the SCH control and prevention programs are centrally designed and intended to be implemented along with MDA across the country [4], this review has shown differences in KAP levels depending on geographical settings and study populations. In addition to inadequate health education and promotion, sociocultural factors could also pose barriers to changing attitudes and practices regarding SCH control and prevention [15]. According to this review, there is a lack of comprehensive knowledge about sources of infection, routes and modes of transmission, morbidity, treatment and prevention of SCH in Ethiopia. This is unfortunate as health promotion for infectious diseases of public health importance has been carried out under the Health Extension Program since 2004–2005 [6]. Furthermore, SCH was identified as one of the five chemotherapy-preventable NTDs in Ethiopia. Chemotherapy was accompanied by health education both in school and in the community [4]. The lower level of knowledge could be due to the inadequacy of health education sessions. For example, we observed that school and community-based education sessions were only conducted at the time of deworming, which is an inadequate method to gain good knowledge and change community behavior. When properly implemented, health education has a high input-output ratio and is a cost-effective prevention and control method. Comprehensive knowledge contributes to a positive attitude, which in turn contributes to behavioral changes such as reducing risky activities [27]. However, behavior change will only occur if health education is accompanied by safe water supplies and basic sanitation [28, 29]. This issue was identified in this review, where participation in risky activities (frequent water contact) was high regardless of participants' level of knowledge [17–26]. Misconceptions about SCH transmission and prevention as well as low levels of knowledge can

lead to poor prevention practices [30, 31]. In Ethiopia, there are both MDA and health education programs for SCH and STHs are implemented together. We understood that there is confusion regarding the transmission and prevention of SCH and STHs [17-19, 24, 25]. For example, in one study, participants believed that consuming contaminated food (23.7%), dirty hands (16.2%), and playing with soil (20%) predisposed to infection [24]; although these actually predispose to STH infection rather than SCH infection. Therefore, the dissemination of the correct information specific to each disease should be particularly emphasized as part of health education in programs that include the control of more than one parasite (since it concerns STH together with SCH) and will be a key to To enable people to apply appropriate preventive measures [32].

Misunderstandings due to false or mixed messages can lead to false perceptions/beliefs in the community [33], which in turn impact their health behaviors and participation in infection prevention [34]. Differences in health education implementation in different transmission settings and study populations could lead to different behavioral changes in Ethiopia. For example, in two studies, the majority, namely 94% [19] and 66.7% [17] of aware participants agreed that SCH is preventable, whereas 89.6% [21] and 79% [20] in other studies did not agree with this. There were also differences in attitudes towards at-risk populations, severity, treatment and prevention across different studies [17–26]. The real purpose of health education is to impart knowledge about SCH and convince the community of their commitment to disease prevention and control. However, in the reviewed studies, the majority of participants were involved in risky activities, regardless of their knowledge and attitudes toward SCH [17–26]. This could be due to the fact that health education was not complemented by basic WASH facilities and health services [15].

Limitations

The proportion of participants with good knowledge, positive attitudes and good prevention practices would have been quantified based on the average scores of the questionnaire items using Bloom's modified cutoff points [35]. We were unable to do this because the items used to assess the KAP showed large differences and were therefore missing. Standard definition of knowledge, attitude and practice. Therefore, the interpretation of the KAP results was subjective. This also made it difficult to compare KAP values between study results. There was wide variation in the depth of data presented in the reviewed studies, such that studies that presented more data were overrepresented in the review. Due to the limited number of studies, it was difficult to draw definitive conclusions about the KAP value in Ethiopia.

Conclusions

The results of the reviewed studies showed that people's KAP scores were inadequate despite both community and school-based health education platforms having been established and implemented for more than a decade. This brings to mind the future strengthening of the health education program and the monitoring of the practical implementation and impact assessment of the existing programs. Furthermore, the joint implementation of two programs (namely STH and SCH) should be precise enough to avoid confusion and mixed information. Preparation of a standard context-specific KAP assessment tool could be the direct responsibility of the FMoH NTD Prevention and Control Program. In addition, further studies at both school and community

levels are recommended to examine KAP levels and associated factors in different endemic areas of all vulnerable populations.
